# Upregulation of TGF-β-induced HSP27 by HSP90 inhibitors in osteoblasts

**DOI:** 10.1186/s12891-022-05419-1

**Published:** 2022-05-26

**Authors:** Gen Kuroyanagi, Haruhiko Tokuda, Kazuhiko Fujita, Tetsu Kawabata, Go Sakai, Woo Kim, Tomoyuki Hioki, Junko Tachi, Rie Matsushima-Nishiwaki, Takanobu Otsuka, Hiroki Iida, Osamu Kozawa

**Affiliations:** 1 Department of Orthopedic Surgery, Nagoya City University Graduate School of Medical Sciences, 1, Kawasumi, Mizuho-cho, Mizuho-ku, Nagoya, 467-8601 Japan; 2 Department of Rehabilitation Medicine, Nagoya City University Graduate School of Medical Sciences, Nagoya, Japan; 3 Department of Pharmacology, Gifu University Graduate School of Medicine, Gifu, Japan; 4 Department of Clinical Laboratory/Medical Genome Center Biobank, National Center for Geriatrics and Gerontology, Obu, Japan; 5 Department of Metabolic Research, National Center for Geriatrics and Gerontology, Obu, Japan; 6 Department of Anesthesiology and Pain Medicine, Gifu University Graduate School of Medicine, Gifu, Japan; 7 Department of Dermatology, Kizawa Memorial Hospital, Minokamo, Japan

**Keywords:** Heat shock protein, HSP90, HSP90 inhibitor, HSP27, TGF-β, SAPK/JNK, Osteoblast

## Abstract

**Background:**

Heat shock protein (HSP) 90 functions as a molecular chaperone and is constitutively expressed and induced in response to stress in many cell types. We have previously demonstrated that transforming growth factor-β (TGF-β), the most abundant cytokine in bone cells, induces the expression of HSP27 through Smad2, p44/p42 mitogen-activated protein kinase (MAPK), p38 MAPK, and stress-activated protein kinase/c-Jun N-terminal kinase (SAPK/JNK) in mouse osteoblastic MC3T3-E1 cells. This study investigated the effects of HSP90 on the TGF-β-induced HSP27 expression and the underlying mechanism in mouse osteoblastic MC3T3-E1 cells.

**Methods:**

Clonal osteoblastic MC3T3-E1 cells were treated with the HSP90 inhibitors and then stimulated with TGF-β. HSP27 expression and the phosphorylation of Smad2, p44/p42 MAPK, p38 MAPK, and SAPK/JNK were evaluated by western blot analysis.

**Result:**

HSP90 inhibitors 17-dimethylaminoethylamino-17-demethoxy-geldanamycin (17-DMAG) and onalespib significantly enhanced the TGF-β-induced HSP27 expression. TGF-β inhibitor SB431542 reduced the enhancement by 17-DMAG or onalespib of the TGF-β-induced HSP27 expression levels. HSP90 inhibitors, geldanamycin, onalespib, and 17-DMAG did not affect the TGF-β-stimulated phosphorylation of Smad2. Geldanamycin did not affect the TGF-β-stimulated phosphorylation of p44/p42 MAPK or p38 MAPK but significantly enhanced the TGF-β-stimulated phosphorylation of SAPK/JNK. Onalespib also increased the TGF-β-stimulated phosphorylation of SAPK/JNK. Furthermore, SP600125, a specific inhibitor for SAPK/JNK, significantly suppressed onalespib or geldanamycin’s enhancing effect of the TGF-β-induced HSP27 expression levels.

**Conclusion:**

Our results strongly suggest that HSP90 inhibitors upregulated the TGF-β-induced HSP27 expression and that these effects of HSP90 inhibitors were mediated through SAPK/JNK pathway in osteoblasts.

**Supplementary Information:**

The online version contains supplementary material available at 10.1186/s12891-022-05419-1.

## Background

Heat shock proteins (HSPs), abundantly expressed in many cell types, are induced in response to stressful conditions such as heat stress and pathological conditions [1]. HSPs are recognized as molecular chaperones and help the folding of nascent proteins and the refolding of denatured proteins [1]. Based on the molecular sizes, HSPs are generally divided into seven major groups such as HSPH (HSP110), HSPC (HSP90), HSPA (HSP70), HSPD/E (HSP60/HSP10), CCT (TRiC), DNAJ (HSP40), and HSPB (small molecular size HSPs) [1, 2]. HSP27 is a major protein in the small molecular size HSPs and works independently of ATP [1]. As an ATP-independent molecular chaperone, HSP27 binds to misfolded proteins and transfers them to the ATP-dependent chaperones, including HSP90 and HSP70 for protein refolding or to proteasomes for protein degradation [1, 3]. Although HSP27 exists typically in the large oligomer, the conformational change to the monomer or the dimers occurs when it is phosphorylated [1]. Conversely, HSP90 is one of the most abundant proteins in human cells, comprising 1–2% of cellular proteins under physiological conditions and 4–6% under stressful conditions [3, 4]. HSP90 is also known as an ATP-dependent molecular chaperone and plays central roles in stabilizing and activating the client proteins [5]. HSP90, as a molecular chaperone, participates in stabilizing and functioning numerous oncogenic signaling proteins in cancer, including breast and lung cancers [5–7]. HSP90 expression is markedly increased in cancer specimens compared to the normal tissues [4, 8]. Thus, inhibition of HSP90 function using an HSP90 inhibitor is now considered a therapeutic modality in treating specific cancers [4, 8]. Using clinical trials, accumulating evidence suggests that the HSP90 inhibitors such as geldanamycin, 17-allylamino-17-demethoxy-geldanamycin (17-AAG), and 17-dimethylaminoethylamino-17-demethoxy-geldanamycin (17-DMAG) can be used for the treatment of cancer disease [7]. Also, HSP90 inhibitors have been proposed as a novel class of senolytics to reduce age-related symptoms in vivo [9].

During bone remodeling, the continuous process of renewal throughout human life, bone resorption by osteoclasts is followed by bone formation by osteoblasts [10, 11]. For retaining the volume and the strength, the process is finely balanced with coupling to ensure sufficient new bone formation at the resorption area [10, 11]. In contrast, under pathological conditions such as osteoporosis with aging, bone resorption exceeds formation, resulting in bone loss and an increased risk of osteoporotic fractures [10, 11]. Transforming growth factor-β (TGF-β), a member of TGF-β superfamily consists of bone morphogenic proteins and activin, is the most abundant cytokine in bone cells and plays a crucial role in bone remodeling [12]. TGF-β embedded in the bone matrix is released when osteoclasts activate bone resorption and recruit osteoblast precursors to start bone formation [12]. Regarding the signaling mechanisms, TGF-β activates Smad signaling pathways, including Smad2 and Smad3 [13], and non-Smad pathways such as p44/p42 mitogen-activated protein kinase (MAPK), p38 MAPK, and stress-activated protein kinase/c-Jun N-terminal kinase (SAPK/JNK) [14]. Our previous studies have shown that TGF-β induces the expression of HSP27 through Smad2, p44/p42 MAPK, p38 MAPK, and SAPK/JNK in mouse osteoblastic MC3T3-E1 cells [15, 16].

Although HSPs functions in osteoblasts have not yet been clarified, we have already demonstrated that HSP27 down-regulates the migration of mouse osteoblastic MC3T3-E1 cells induced by platelet derived growth factor-BB (PDGF-BB) [17]. We have also demonstrated that HSP27 in unphosphorylated form has an inhibitory effect on osteocalcin release, while it has a stimulatory effect on mineralization in osteoblasts [18]. Regarding the HSP90 function in osteoblasts, bisphosphonates, a therapeutic tool for osteoporosis, and low-intensity pulsed ultrasound stimulation (LIPUS), a clinically used device for accelerating bone fracture healing, could reportedly induce HSP90 expression in osteoblastic cells [19, 20]. We have already demonstrated that HSP90 inhibitors upregulate the endothelin-1-induced HSP27 expression through the SAPK/JNK pathway but not p38 MAPK in mouse osteoblastic MC3T3-E1 cells [21], and that HSP90 inhibitors enhance the prostaglandin D_2_ (PGD_2_)-induced HSP27 expression through both the SAPK/JNK and p38 MAPK pathways in these cells [22]. However, the mechanism whereby HSP90 functions on the expression of HSP27 in osteoblasts remains unclear.

In this study, we investigated the effects of HSP90 inhibitors on the TGF-β-induced HSP27 expression and the underlying mechanism using mouse osteoblastic MC3T3-E1 cells. We identified that HSP90 inhibitors upregulated the TGF-β-induced HSP27 expression and that the effects were mediated through the SAPK/JNK pathway in osteoblasts.

## Methods

### Materials

TGF-β was purchased from R&D Systems, Inc. (Minneapolis, MN, USA). Onalespib was purchased from Selleckchem (Houston, TX, USA). Geldanamycin was obtained from Sigma-Aldrich Co. (St. Louis, MO, USA). 17-DMAG and SP600125 were purchased from Calbiochem-Novabiochem Co. (La Jolla, CA, USA). SB431542 was purchased from Funakoshi Co., Ltd. (Tokyo, Japan). HSP27 antibodies, phosphorylated HSP27 antibodies, and glyceraldehyde-3-phosphate dehydrogenase (GAPDH) antibodies were obtained from Santa Cruz Biotechnology, Inc. (Santa Cruz, CA, USA). Phospho-specific Smad2, Smad2, phospho-specific p44/p42 MAPK, p44/p42 MAPK, phospho-specific p38 MAPK antibodies, p38 MAPK antibodies, phospho-specific SAPK/JNK antibodies, and SAPK/JNK antibodies, were obtained from Cell Signaling Technology, Inc. (Beverly, MA, USA). An ECL Western blotting detection system was obtained from GE Healthcare Life Sciences (Chalfont, UK). Other materials and chemicals were obtained from commercial sources. Onalespib, geldanamycin, 17-DMAG, SP600125, and SB431542 were dissolved in dimethyl sulfoxide (DMSO). The maximum concentration of DMSO was 0.1%, which did not affect the assay for Western blot analysis [21–23].

### Cell culture

Cloned osteoblastic MC3T3-E1 cells were generously provided by Dr. M. Kumegawa (Meikai University, Sakado, Japan). The osteoblastic MC3T3-E1 cells, established from neonatal mouse calvaria [24], were incubated at 37 °C with 5% CO_2_ and cultured in α-minimum essential medium (α-MEM) supplemented with 10% fetal bovine serum (FBS) as previously described [25]. Cells were seeded into 90-mm diameter dishes (2 × 10^5^ cells/dish) in α-MEM supplemented with 10% FBS. α-MEM medium was supplemented with 0.3% FBS after 5 days. After 48 hours, the cells were used for the experiments. Cells of passages under 20 were selected for the experiments.

### Western blot analysis

The cultured osteoblasts were pre-incubated with 10 μM of SP600125, 3 μM of SB431542 or vehicle for 60 min. The cells were subsequently pretreated with various doses of 17-DMAG, onalespib, or geldanamycin for 60 min and then treated by 3 or 10 ng/ml of TGF-β or vehicle in α-MEM supplemented with 0.3% FBS. As previously described [21–23], the osteoblastic cells were incubated for the indicated periods and washed twice in phosphate-buffered saline (PBS). In brief, lysate containing 62.5 mM of Tris/HCl, pH 6.8, 2% sodium dodecyl sulfate (SDS), 50 mM of dithiothreitol, and 10% glycerol was used to extract total protein from the cells [21–23]. The cells were also homogenized and sonicated in the lysate buffer. Proteins were separated by SDS-polyacrylamide gel electrophoresis (PAGE) using Laemmli’s method [26] in 10% polyacrylamide gels and transferred to polyvinylidene difluoride (PVDF) membranes (Bio-Rad Laboratories, Inc., Hercules, CA, USA). Following blocking for 1 h at room temperature in 5% fat free dry milk in Tris-buffered saline-Tween (TBS-T; 20 mM of Tris-HCl, pH 7.6, 137 mM of NaCl and 0.1% Tween 20), membranes were incubated more than 12 h at 4 °C with the following primary antibodies (HSP27 antibodies (sc-1049); phospho-specific Smad2 antibodies (#3108); Smad2 antibodies (#3102); phospho-specific p44/p42 MAPK antibodies (#9101); p44/p42 MAPK antibodies (#9102); phospho-specific p38 MAPK antibodies (#4511); p38 MAPK antibodies (#9212); phospho-specific SAPK/JNK antibodies (#4668); SAPK/JNK antibodies (#9252) and GAPDH antibodies (#60004–1-IG)). The PVDF membranes were washed three times in TBS-T. The membranes were then incubated with a secondary antibody (goat against rabbit IgG) with 5% fat free dry milk in TBS-T for 1 h at room temperature and washed three times with TBS-T. Protein bands were visualized on X-ray film by ECL Western blotting detection system. We showed full length Western blotting images with a molecular marker in the Supplementary Information file. As for the induction of HSP27 in osteoblast-like MC3T3-E1 cells, we have previously shown that TGF-β significantly induces the expression of HSP27 at 12 h after the stimulation [15, 16]. Conversely, regarding the intracellular signaling of TGF-β, we have also reported that TGF-β significantly induces the phosphorylation of Smad2, p44/p42 MAPK, p38 MAPK, and SAPK/JNK at 2 h after the stimulation [15]. Therefore, in this study, we conducted the experiments about HSP27 induction in a long time point (12 h) and intracellular signaling in a short time point (2 h). The controls included the same amount of 0.1% of DMSO in this study. The vehicle for the TGF-β treatment was a solvent containing PBS, in which TGF-β was dissolved.

Regarding the concentration of HSP90 inhibitors, we previously investigated the effects of 10 and 20 nM of 17-DMAG and 10, 20 and 30 nM of onalespib on the expression levels of HSP27 induced by endothelin-1 (ET-1) in osteoblast-like MC3T3-E1 cells [21]. Thus, we adopted same concentrations of HSP90 inhibitors for the induction of HSP27 experiments. On the other hand, we also reported the effects of 0.3, 0.7 and 1.0 μM of geldanamycin, onalespib and 17-DMAG on the phosphorylation of p38 MAP kinase induced by ET-1 in osteoblast-like MC3T3-E1 cells [21]. Therefore, we herein adopted the condition (0.3, 0.7 and 1.0 μM of geldanamycin and 17-DMAG; 0.3 and 1.0 μM of onalespib) similar to the previous study for the signaling experiments in this study.

### Densitometric analysis

Densitometric analysis for Western blotting was done by a scanner and ImageJ ver. 1.49 software (NIH, Bethesda, MD, USA). Phosphorylation levels were assessed by the following method. The background-subtracted signal intensity of each phosphorylation signal was normalized to the respective intensity of total protein and plotted as the fold increase compared to that in the control cells without stimulation. Regarding the quantification of HSP27 expression levels, the signal intensity of each HSP27 band was normalized to the respective intensity of GAPDH bands.

### Statistical analysis

All experiments were repeated at least three times using three independent cell preparations. Three separate dishes of cells plated at three independent experiments were performed on different days. All data were presented as the mean ± standard error of the mean (SEM) of triplicate experiment results. Differences between groups were determined by an analysis of variance followed by the Bonferroni method for multiple comparisons between pairs. *P* < 0.05 was set to indicate a statistically significant difference.

## Results

### HSP90 inhibitor 17-DMAG upregulates TGF-β-induced HSP27 expression in osteoblastic MC3T3-E1 cells

**Fig. 1:**
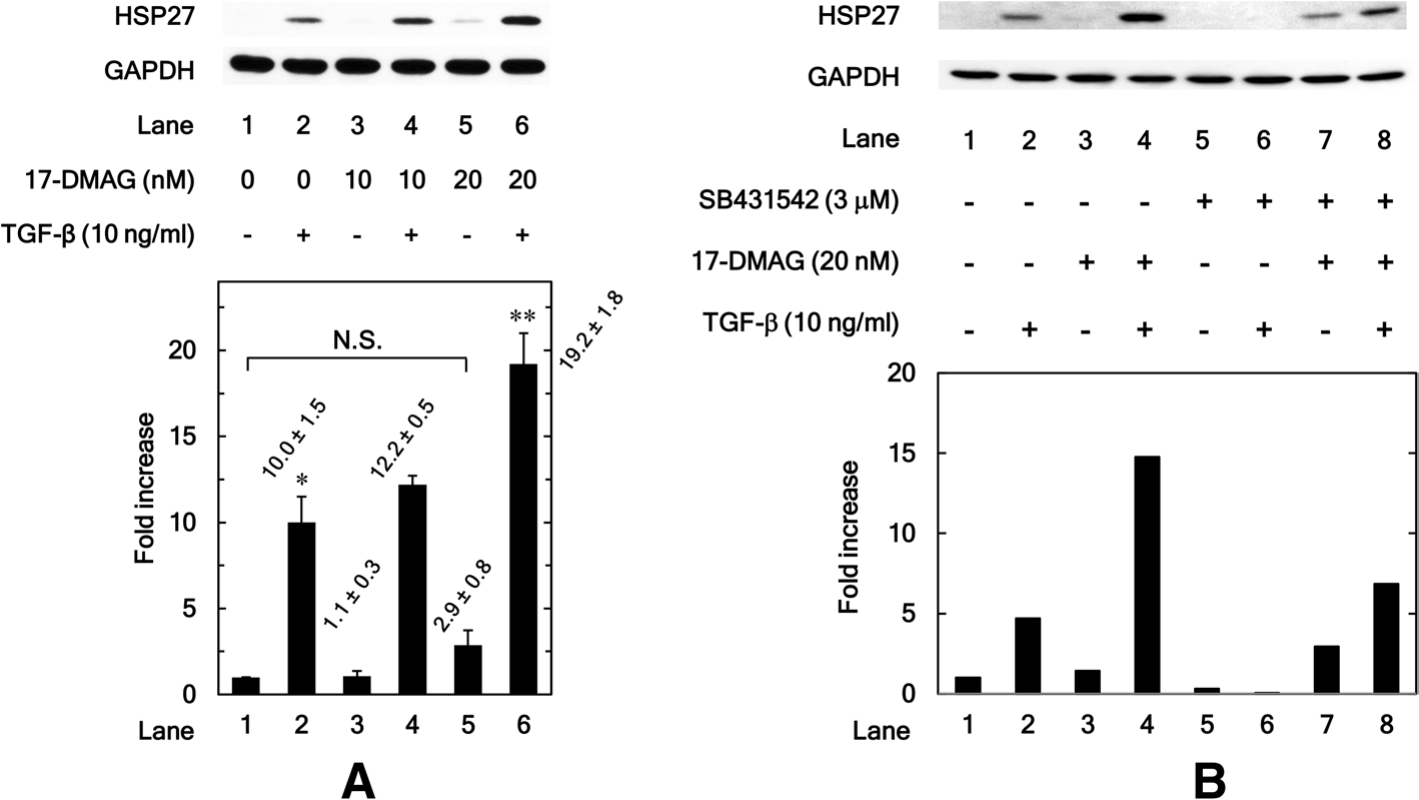
**A** HSP90 inhibitor 17-DMAG upregulates TGF-β-induced HSP27 expression in osteoblastic MC3T3-E1 cells. The cultured osteoblasts were pretreated with 10 or 20 nM of 17-DMAG for 60 min, and subsequently incubated by 10 ng/ml of TGF-β or vehicle for 12 h. Cell extracts were analyzed by SDS-PAGE and Western blotting using antibodies of HSP27 or GAPDH. The histogram shows the quantitative representations of the levels of HSP27 normalized with each GAPDH gained from laser densitometric analysis. The levels were expressed as the fold increase to the basal levels presented as lane 1. Triplicate determinations of Western blot analysis were performed corresponding to three independent cell preparations. Each value represents the mean ± S.E.M. of triplicate determinations from three independent cell preparations. *P < 0.05, compared to the value of the control cells without TGF-β-stimulation. **P < 0.05, compared to the value of TGF-β alone. **B** TGF-β inhibitor SB431542 reduces the enhancement by 17-DMAG of the TGF-β-induced HSP27 expression in MC3T3-E1 cells. The cultured osteoblasts were pre-incubated with 3 μM of SB431542 or vehicle for 60 min. The cells were subsequently pretreated with 20 nM of 17-DMAG or vehicle for 60 min, and then stimulated by 10 ng/ml of TGF-β or vehicle for 12 h. Cell extracts were analyzed by SDS-PAGE and Western blotting using antibodies of HSP27 or GAPDH. The histogram shows the quantitative representations of the levels of HSP27 normalized with each GAPDH gained from laser densitometric analysis. The levels were expressed as the fold increase to the basal levels presented as lane 1

We have previously demonstrated that TGF-β strongly induced HSP27 expression in osteoblastic MC3T3-E1 cells [15, 16]. To investigate the effects of HSP90 on the HSP27 expression in osteoblasts, we first examined whether HSP90 inhibitor 17-DMAG [27] affects the TGF-β-induced HSP27 expression levels in mouse osteoblastic MC3T3-E1 cells. We found that treatment with 20 nM of 17-DMAG significantly enhanced the TGF-β-induced HSP27 expression in MC3T3-E1 cells (*P* = 0.02) (Fig. 1A). We also found that 20 nM of 17-DMAG tended to increase HSP27 expression without TGF-β stimulation, but there was no significant difference (Comparison of lane 5 to lane 1: *P* = 0.09; Comparison of lane 5 to lane 3: *P* = 0.11).

### TGF-β inhibitor SB431542 reduces the enhancement by 17-DMAG of the TGF-β-induced HSP27 expression in MC3T3-E1 cells

Furthermore, we used TGF-β inhibitor SB431542 to eliminate the confounding effects of endogenous TGF-β to actually elucidate whether enhancement of HSP27 in response to HSP90 inhibition is dependent on TGF-β or not. As a result, SB431542 reduced the enhancement by 17-DMAG of the TGF-β-induced HSP27 expression levels (Fig. 1B).

### HSP90 inhibitor onalespib upregulates TGF-β-induced HSP27 expression in MC3T3-E1 cells

**Fig. 2:**
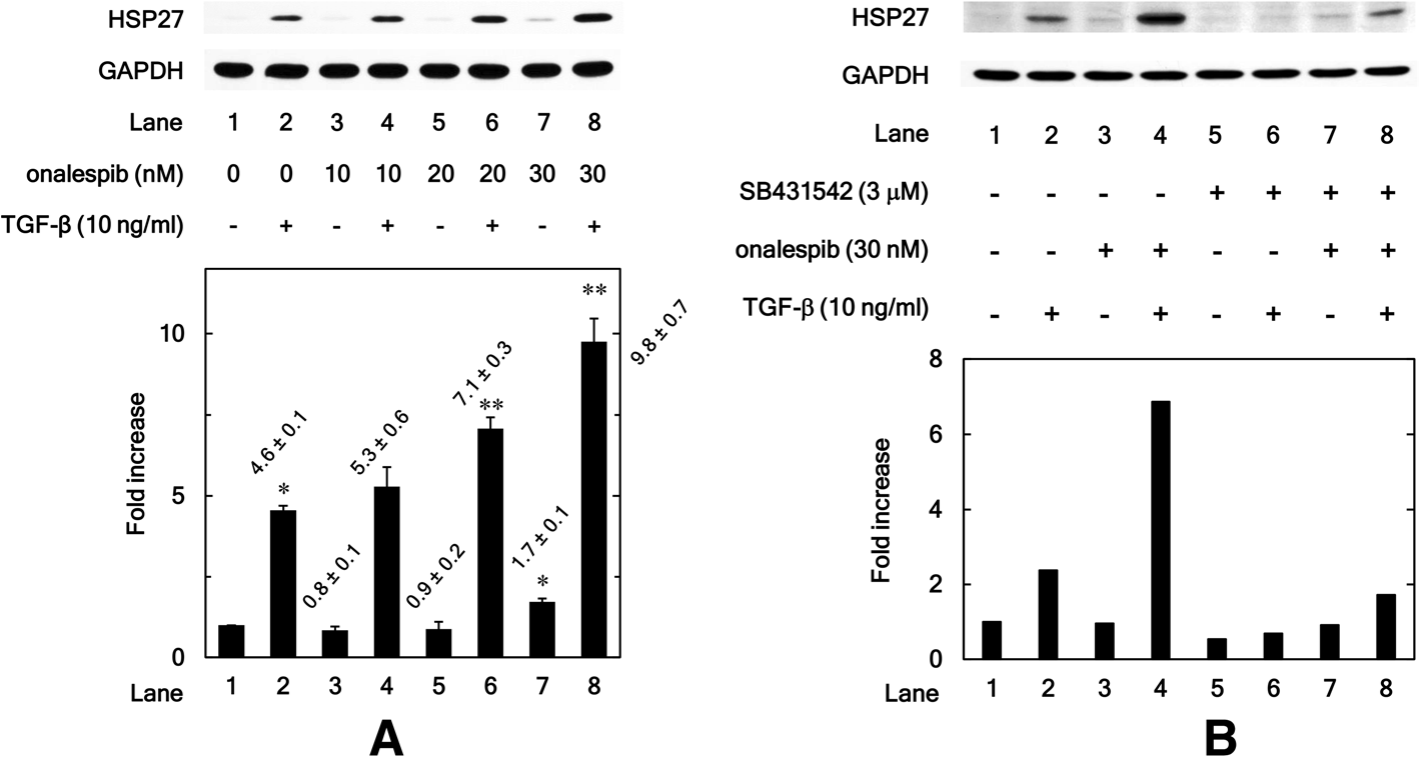
**A** HSP90 inhibitor onalespib upregulates TGF-β-induced HSP27 expression in MC3T3-E1 cells. The cultured osteoblasts were pretreated with 10, 20 or 30 nM of onalespib for 60 min, and subsequently incubated by 10 ng/ml of TGF-β or vehicle for 12 h. Cell extracts were analyzed by SDS-PAGE and Western blotting using antibodies of HSP27 or GAPDH. The histogram shows the quantitative representations of the levels of HSP27 normalized with each GAPDH gained from laser densitometric analysis. The levels were expressed as the fold increase to the basal levels presented as lane 1. Triplicate determinations of Western blot analysis were performed corresponding to three independent cell preparations. Each value represents the mean ± S.E.M. of triplicate determinations from three independent cell preparations. *P < 0.05, compared to the value of the control cells without TGF-β-stimulation. **P < 0.05, compared to the value of TGF-β alone. **B** TGF-β inhibitor SB431542 reduces the enhancement by onalespib of the TGF-β-induced HSP27 expression in MC3T3-E1 cells. The cultured osteoblasts were pre-incubated with 3 μM of SB431542 or vehicle for 60 min. The cells were subsequently pretreated with 30 nM of onalespib or vehicle for 60 min, and then stimulated by 10 ng/ml of TGF-β or vehicle for 12 h. Cell extracts were analyzed by SDS-PAGE and Western blotting using antibodies of HSP27 or GAPDH. The histogram shows the quantitative representations of the levels of HSP27 normalized with each GAPDH gained from laser densitometric analysis. The levels were expressed as the fold increase to the basal levels presented as lane 1

Using onalespib, which is structurally distinct from 17-DMAG but is also ATP-competitive inhibitor [28], we further examined HSP90 effects on HSP27 expression in MC3T3-E1 cells. Similar to 17-DMAG, onalespib alone did not affect HSP27 expression levels but significantly enhanced the levels of TGF-β-induced HSP27 (20 nM: *P* = 0.002; 30 nM: *P* = 0.002) (Fig. 2A). We found that the onalespib’s significant effect on HSP27 induction was observed between the 20 and 30 nM ranges. On the other hand, 30 nM of onalespib significantly enhanced HSP27 expression without TGF-β stimulation (Comparison of lane 7 to lane 1: *P* = 0.002; Comparison of lane 7 to lane 5: *P* = 0.03).

### TGF-β inhibitor SB431542 reduces the enhancement by onalespib of the TGF-β-induced HSP27 expression in MC3T3-E1 cells

We also found that SB431542 decreased the enhancement by onalespib of the TGF-β-induced HSP27 expression levels (Fig. 2B).

### HSP90 inhibitors, geldanamycin, onalespib, and 17-DMAG do not affect the phosphorylation of Smad2 induced by TGF-β in osteoblastic MC3T3-E1 cells

**Fig. 3:**
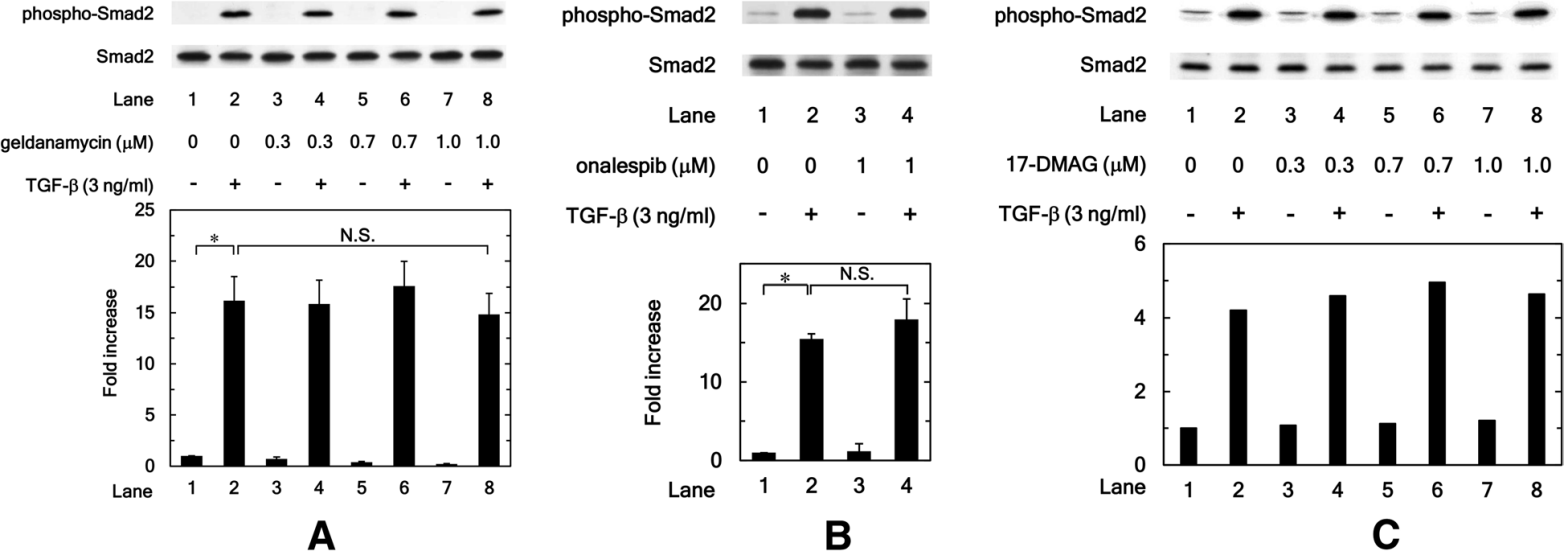
HSP90 inhibitors, geldanamycin, onalespib and 17-DMAG do not affect the phosphorylation of Smad2 induced by TGF-β in osteoblastic MC3T3-E1 cells. The cultured osteoblasts were pretreated with 0.3, 0.7 or 1.0 μM of geldanamycin (**A**), 1 μM of onalespib (**B**), 0.3, 0.7 or 1.0 μM of 17-DMAG (**C**) or vehicle for 60 min, and then incubated by 3 ng/ml of TGF-β or vehicle for 60 min. Cell extracts were analyzed by SDS-PAGE and Western blotting using antibodies of phospho-specific Smad2 or Smad2. The histogram shows the quantitative representations of phosphorylated Smad2 normalized with each total Smad2 gained from laser densitometric analysis. The levels were expressed as the fold increase to the basal levels presented as lane 1. **A, B **Triplicate determinations of Western blot analysis were performed corresponding to three independent cell preparations. Each value represents the mean ± S.E.M. of triplicate determinations from three independent cell preparations. *P < 0.05, compared to the value of the control cells without TGF-β-stimulation. N.S. means no significant difference between the indicated pairs

Regarding the canonical pathways, it has been established that Smad2 and Smad3 phosphorylation is firstly required for TGF-β signal transduction [13]. Our previous study has shown that TGF-β stimulates Smad2 phosphorylation in osteoblastic MC3T3-E1 cells [29]. Thus, we next examined whether HSP90 inhibitors affect the Smad2 phosphorylation induced by TGF-β. The results showed that geldanamycin, the first HSP90 inhibitor characterized as naturally occurring [30], did not affect the Smad2 phosphorylation with (0.3 μM: *P* = 0.93; 0.7 μM: *P* = 0.69; 1.0 μM: *P* = 0.68) or without TGF-β stimulation (Fig. 3A). Additionally, onalespib and 17-DMAG also did not affect Smad2 phosphorylation with (Onalespib: *P* = 0.41) or without TGF-β stimulation in MC3T3-E1 cells (Fig. 3B and C).

### Geldanamycin does not affect the phosphorylation of p44/p42 MAPK or p38 MAPK induced by TGF-β in osteoblastic MC3T3-E1 cells

**Fig. 4:**
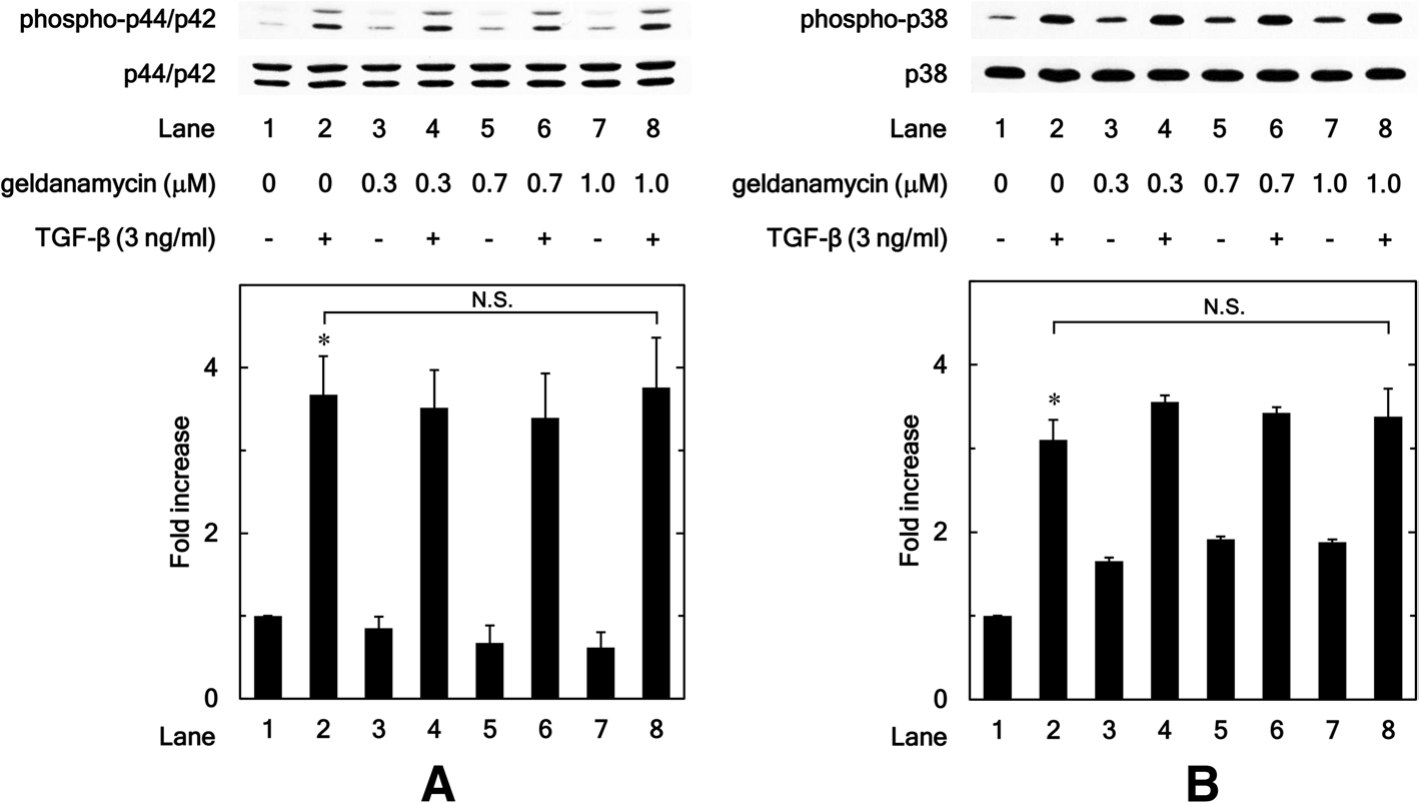
Geldanamycin does not affect the phosphorylation of p44/p42 MAPK or p38 MAPK induced by TGF-β in osteoblastic MC3T3-E1 cells. The cultured osteoblasts were pretreated with 0.3, 0.7 or 1.0 μM of geldanamycin for 60 min, and then incubated by 3 ng/ml of TGF-β or vehicle for 2 h. Cell extracts were analyzed by SDS-PAGE and Western blotting using antibodies of phospho-specific p44/p42 MAPK or p44/p42 MAPK (**A**) or phospho-specific p38 MAPK or p38 MAPK (**B**). The histogram shows the quantitative representations of phosphorylated p44/p42 MAPK normalized with each total p44/p42 MAPK (**A**) or the levels of phosphorylated p38 MAPK normalized with each total p38 MAPK (**B**) gained from laser densitometric analysis. The levels were expressed as the fold increase to the basal levels presented as lane 1. Triplicate determinations of Western blot analysis were performed corresponding to three independent cell preparations. Each value represents the mean ± S.E.M. of triplicate determinations from three independent cell preparations. N.S. means no significant difference between the indicated pairs

In addition to the canonical pathways, it is well recognized that TGF-β can activate various other intracellular signaling pathways called non-canonical pathways, including MAPKs [14]. Our previous studies have shown that TGF-β induces HSP27 expression through the p44/p42 MAPK, p38 MAPK, and SAPK/JNK pathways in mouse osteoblastic MC3T3-E1 cells [15, 16]. Therefore, we examined whether geldanamycin affects p44/p42 MAPK phosphorylation induced by TGF-β, and found that geldanamycin did not affect p44/p42 MAPK phosphorylation with (0.3 μM: *P* = 0.82; 0.7 μM: *P* = 0.72; 1.0 μM: *P* = 0.91) or without TGF-β stimulation in osteoblastic MC3T3-E1 cells (Fig. 4A). We also examined whether geldanamycin affects p38 MAPK phosphorylation induced by TGF-β, and found that geldanamycin did not affect p38 MAPK phosphorylation with (0.3 μM: *P* = 0.15; 0.7 μM: *P* = 0.26; 1.0 μM: *P* = 0.54) or without TGF-β stimulation (Fig. 4B).

### HSP90 inhibitors, geldanamycin, and onalespib stimulate the phosphorylation of SAPK/JNK induced by TGF-β in osteoblastic MC3T3-E1 cells

**Fig. 5:**
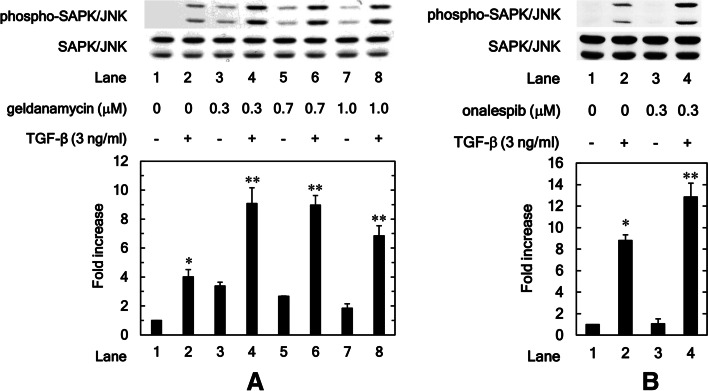
HSP90 inhibitors, geldanamycin and onalespib, stimulate the phosphorylation of SAPK/JNK induced by TGF-β in osteoblastic MC3T3-E1 cells. The cultured osteoblasts were pretreated with 0.3, 0.7, 1.0 μM of geldanamycin (**A**) and 0.3, μM of onalespib (**B**) for 60 min, and then incubated by 3 ng/ml of TGF-β or vehicle for 2 h. Cell extracts were analyzed by SDS-PAGE and Western blotting using antibodies of phospho-specific SAPK/JNK or SAPK/JNK. The histogram shows the quantitative representations of the levels of phosphorylated SAPK/JNK normalized with each total SAPK/JNK gained from laser densitometric analysis. The levels were expressed as the fold increase to the basal levels presented as lane 1. Triplicate determinations of Western blot analysis were performed corresponding to three independent cell preparations. Each value represents the mean ± S.E.M. of triplicate determinations from three independent cell preparations. *P < 0.05, compared to the value of the control cells without TGF-β-stimulation. **P < 0.05, compared to the value of TGF-β alone

We further examined whether HSP90 inhibitor affects SAPK/JNK phosphorylation induced by TGF-β in osteoblastic MC3T3-E1 cells. Contrary to the effects on p44/p42 MAPK and p38 MAPK, we found that geldanamycin significantly enhanced SAPK/JNK phosphorylation induced by TGF-β between 0.3 to 1.0 μM (0.3 μM: *P* = 0.013; 0.7 μM: *P* = 0.004; 1.0 μM: *P* = 0.03) (Fig. 5A). As well as geldanamycin, we also found that onalespib also significantly strengthened the phosphorylation of SAPK/JNK induced by TGF-β (*P* = 0.04) (Fig. 5B).

### SAPK/JNK inhibitor, SP600125, suppresses the enhancing effect by onalespib and geldanamycin of the TGF-β-induced HSP27 expression in osteoblastic MC3T3-E1 cells

**Fig. 6:**
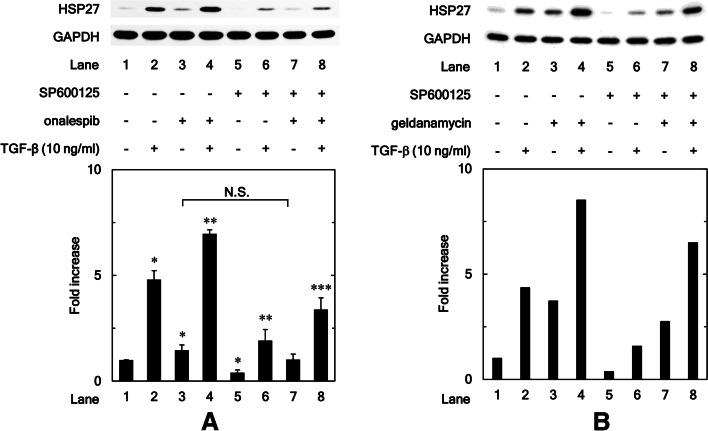
SAPK/JNK inhibitor, SP600125, suppresses the enhancing effect by onalespib and geldanamycin of the TGF-β-induced HSP27 expression in osteoblastic MC3T3-E1 cells. The cultured osteoblasts were pre-incubated with 10 μM of SP600125 or vehicle for 60 min. The cells were subsequently pretreated with 30 nM of onalespib (**A**), 30 nM of geldanamycin (**B**) or vehicle for 60 min, and then stimulated by 10 ng/ml of TGF-β or vehicle for 12 h. Cell extracts were analyzed by SDS-PAGE and Western blotting using antibodies of HSP27 or GAPDH. The histogram shows the quantitative representations of HSP27 levels normalized with each GAPDH gained from laser densitometric analysis. The levels were expressed as the fold increase to the basal levels presented as lane 1. **A** Triplicate determinations of Western blot analysis were performed corresponding to three independent cell preparations. Each value represents the mean ± S.E.M. of triplicate determinations from three independent cell preparations. *P < 0.05, compared to the value of the control cells without TGF-β-stimulation. **P < 0.05, compared to the value of TGF-β alone. ***P < 0.05, compared to the value of TGF-β and onalespib treatment

To examine the involvement of SAPK/JNK in the enhancement by HSP90 inhibitor of HSP27 expression, we investigated SP600125 effects, an inhibitor for SAPK/JNK [31], on the amplification of the TGF-β-induced HSP27 expression in osteoblastic MC3T3-E1 cells by onalespib. We found that SP600125 significantly inhibited the enhancement by onalespib of the TGF-β-induced HSP27 expression levels (*P* = 0.004) (Fig. 6A). On the other hand, SP600125 significantly reduced HSP27 expression in osteoblast-like MC3T3-E1 cells compared to the unstimulated cells (Comparison of lane 5 to lane 1: *P* = 0.01). On the other hand, SP600125 did not significantly reduce the enhancement by onalespib of HSP27 expression levels (Comparison of lane 7 to lane 3: *P* = 0.17). Therefore, SAPK/JNK pathway seems to be involved in HSP27 expression in TGF-β-unstimulated osteoblast-like MC3T3-E1 cells.

We also found that SP600125 suppressed the enhancement by geldanamycin of the TGF-β-induced HSP27 expression levels (Fig. 6B).

## Discussions

In the present study, HSP90’s effects on the TGF-β-induced HSP27 expression were investigated using mouse osteoblastic MC3T3-E1 cells. HSP90 normally exists in many cell types, including osteoblasts [19]. We first demonstrated that HSP90 inhibition using HSP90 inhibitors such as 17-DMAG and onalespib significantly upregulated the TGF-β-induced HSP27 expression in osteoblastic MC3T3-E1 cells. As 17-DMAG and onalespib could diminish the HSP90 regulation to the TGF-β-stimulated event, it is likely that HSP90 negatively regulates the TGF-β-stimulated HSP27 induction in osteoblastic MC3T3-E1 cells. In addition, we used TGF-β inhibitor SB431542 to eliminate the confounding impact of endogenous TGF-β to truly elucidate whether the HSP27 enhancement in response to HSP90 inhibition is TGF-β dependent. As a result, we found that SB431542 reduced the enhancement by 17-DMAG of the TGF-β-induced HSP27 expression levels. In addition, SB431542 decreased the enhancement by onalespib of the TGF-β-induced HSP27 expression levels. Thus, it seems likely that HSP27 enhancement in response to HSP90 inhibition is TGF-β dependent in osteoblast-like MC3T3-E1 cells. On the other hand, we also found that 20 nM of 17-DMAG tended to increase HSP27 expression without TGF-β stimulation. Furthermore, 30 nM of onalespib significantly stimulated HSP27 expression without TGF-β stimulation. Based on these findings, it is likely that HSP90 inhibition may upregulate HSP27 induction in osteoblastic MC3T3-E1 cells without TGF-β stimulation. It has been reported that HSP90 inhibition by 17AAG stimulates HSP27 expression in human melanoma cells [32]. Thus, it is probable that HSP90 inhibition induces HSP27 expression in both TGF-β-dependent and TGF-β-independent pathways in osteoblastic MC3T3-E1 cells.

It has been well known that TGF-β mainly activates two types of signaling pathways, such as the Smad and non-Smad pathways, also called a canonical and non-canonical pathway, respectively [13, 14]. As for the Smad pathway in mouse osteoblastic MC3T3-E1 cells, our previous study showed that TGF-β actually stimulates the phosphorylation of Smad2 [29]. Thus, we examined HSP90 inhibitors’ effects using geldanamycin and onalespib on Smad2 phosphorylation induced by TGF-β in MC3T3-E1 cells. We found that geldanamycin and onalespib hardly affected the TGF-β-stimulated Smad2 phosphorylation, suggesting that TGF-β-stimulated Smad2 activation is unlikely regulated by HSP90 in osteoblasts. Regarding the difference between geldanamycin and 17-DMAG, geldanamycin binds to the ATP binding site of HSP90 and subsequently prevents HSP90 activity as an HSP90 inhibitor. However, due to unacceptable hepatotoxicity, geldanamycin cannot be used in clinical practice [33]. In contrast, 17-DMAG is a semisynthetic derivative of geldanamycin and possesses reduced hepatotoxicity while retaining the molecular activities of geldanamycin [34]. We investigated the effect of 17-DMAG on the phosphorylation of Smad2 induced by TGF-β in MC3T3-E1 cells, and found that 17-DMAG did not affect the phosphorylation of Smad2 with or without TGF-β stimulation in MC3T3-E1 cells. Thus, this result also supports our hypothesis that HSP90 inhibitors do not alter the activation of Smad2 induced by TGF-β in osteoblast-like MC3T3-E1 cells.

Regarding the non-Smad pathway, we have already demonstrated that TGF-β stimulates p44/p42 MAPK, p38 MAPK, and SAPK/JNK phosphorylation in osteoblastic MC3T3-E1 cells [15, 16]. We found that geldanamycin did not affect the TGF-β-stimulated p44/p42 MAPK or p38 MAPK phosphorylation but strongly increased the TGF-β-stimulated SAPK/JNK phosphorylation in these cells. We also confirmed that onalespib significantly enhanced SAPK/JNK phosphorylation stimulated by TGF-β. Thus, the SAPK/JNK activation is probably regulated by HSP90 in the non-canonical pathway of TGF-β in these cells. It is most likely that the upregulation by HSP90 inhibitors of the TGF-β-induced HSP27 expression is mediated by SAPK/JNK, a non-Smad pathway, in osteoblastic MC3T3-E1 cells. However, we do not have data using onalespib on p44/p42 MAPK and p38 MAPK phosphorylation induced by TGF-β. We speculated that p44/p42 MAPK and p38 MAPK might not be involved in the TGF-β-induced HSP27 in MC3T3-E1 cells based on the results treated with geldanamycin. Thus, the experiments treated with onalespib would be necessary to confirm our speculation. We have previously reported that SAPK/JNK acts as a positive regulator in HSP27 induction stimulated by TGF-β in osteoblast-like MC3T3-E1 cells [15, 16]. Thus, the result that SP600125 markedly suppressed the TGF-β-induced HSP27 expression is consistent with our previous reports. In the present study, we showed that SP600125 significantly inhibited the enhancement by onalespib of the TGF-β-induced HSP27 expression levels. Our findings suggest that SP600125 truly functions as a SAPK/JNK inhibitor. Thus, it seems unlikely that SP600125 is a general HSP27 inhibitor of TGF-β-induced HSP27 expression. Our previous study showed that SAPK/JNK and p38 MAPK but not p44/p42 MAPK are involved in the upregulation by HSP90 inhibitors in the PGD_2_-induced HSP27 expression in these cells [22]. We have also demonstrated that SAPK/JNK but not p38 MAPK is involved in enhancing endothelin-1-induced HSP27 expression in these cells by HSP90 inhibitors [21]. Therefore, as far as we know, it is likely that HSP90 regulates HSP27 expression in response to a variety of stimulations at a point upstream of SAPK/JNK commonly in mouse osteoblastic MC3T3-E1 cells. Taking our findings into account, TGF-β and HSP90 inhibition independently stimulate HSP27 expression, and that both HSP90 and TGF-β play a role in the regulation of SAPK/JNK phosphorylation. However, the effect of the interplay between HSP90-TGF-β-SAPK/JNK on the regulation of HSP27 has not been definitively investigated in this study. Further investigation will be necessary to explain the potential effects of TGF-β-independent regulation of HSP27 expression following HSP90 inhibition in osteoblasts.

Although the involvement of HSP90 in bone metabolism is still unclear, bisphosphonates, a group of medicines for osteoporosis, and LIPUS, a device clinically used for non-union and fracture hearing distress, reportedly could induce the HSP90 expression in osteoblasts [19, 20]. In contrast, it has recently been reported that HSP90 inhibition enhances bone formation and rescues glucocorticoid-induced bone loss in mice [35]. Regarding HSP27 function in osteoblasts, we have previously investigated the effect of HSP27 on the bone morphogenetic protein (BMP)-4 or T_3_-induced osteocalcin synthesis in HSP27-transfected MC3T3-E1 cells, and found that BMP-4 or T3-induced osteocalcin levels were markedly lower in HSP27-transfected cells compared to empty vector transfected cells [18]. We have also performed alizarin red staining in MC3T3-E1 cells transfected with the empty vector and HSP27 cDNA vector to examine the role of HSP27 in bone calcification. As a result, the extent of matrix mineralization indicated by alizarin red staining was significantly higher in HSP27-transfected cells than in empty vector transfected cells 19 days after seeding [18]. Thus, HSP27 in the unphosphorylated form upregulates the calcification of mouse osteoblastic MC3T3-E1 cells. In this study, HSP90 inhibition by HSP90 inhibitors enhances HSP27 expression induced by TGF-β in osteoblast-like MC3T3-E1 cells. TGF-β is known to be released from bone matrix in the process of bone resorption, and promote subsequent bone formation [12]. Taking our present findings into account, suppression of HSP90 might likely enhance HSP27 expression induced by TGF-β in the process of bone remodeling, resulting in the upregulation of calcification essential in the osteoblastic bone formation. Our findings might provide a novel therapeutic strategy of HSP90 inhibitors to treat metabolic bone disorders, including osteoporosis or fracture healing disturbance by enhancing HSP27 expression in osteoblasts. Further examination will be needed to investigate the details about HSP90-effect on bone metabolism.

## Conclusions

In summary, our results strongly suggested that HSP90 inhibitors upregulated the TGF-β-induced HSP27 expression and that the effects of HSP90 inhibitors were mediated through the SAPK/JNK pathway in osteoblasts. We believe that HSP90 plays a crucial role in osteoblast function and could be a useful therapeutic target in bone loss disorders, including osteoporosis.

## Supplementary Information

**Additional file 1 .** Figure S1A, S1B, S2A, S2B, S3A, S3B, S3C, S4A, S4B, S5A, S5B, S6A and S6B show full-length gels and blots of Figure 1A, B, 2A 2B, 3A, 3B, 3C, 4A, 4B, 5A, 5B, 6A and 6B, respectively.

## Data Availability

All data generated or analysed during this study are included in this published article and its supplementary information files.

## Abbreviations

HSP: Heat shock protein
TGF-b: Transforming growth factor-b
MAPK: Mitogen-activated protein kinase
SAPK/JNK: Stress-activated protein kinase/c-Jun N-terminal kinase
17-DMAG: 17-dimethylaminoethylamino-17-demethoxy-geldanamycin
PDGF-BB: Platelet derived growth factor-BB
LIPUS: Low-intensity pulsed ultrasound stimulation
PFG2: Prostaglandin D2
GAPDH: Glyceraldehyde-3-phosphate dehydrogenase
DMSO: Dissolved in dimethyl sulfoxide
a-MEM: a-minimum essential medium
FBS: Fetal bovine serum
BMP: Bone morphogenetic protein
PBS: Phosphate-buffered saline
SDS: Sodium dodecyl sulfate
PAGE: Polyacrylamide gel electrophoresis
PVDF: Polyvinylidene difluoride
TBS-T: Tris-buffered saline-Tween
SEM: Standard error of the mean
