# Supplementary figures and images for: Upregulation of TGF-β-induced HSP27 by HSP90 inhibitors in osteoblasts
Source: BMC Musculoskelet Disord. 2022 May 26;23:495. doi: 10.1186/s12891-022-05419-1 (PMC9134601; doi:10.1186/s12891-022-05419-1)

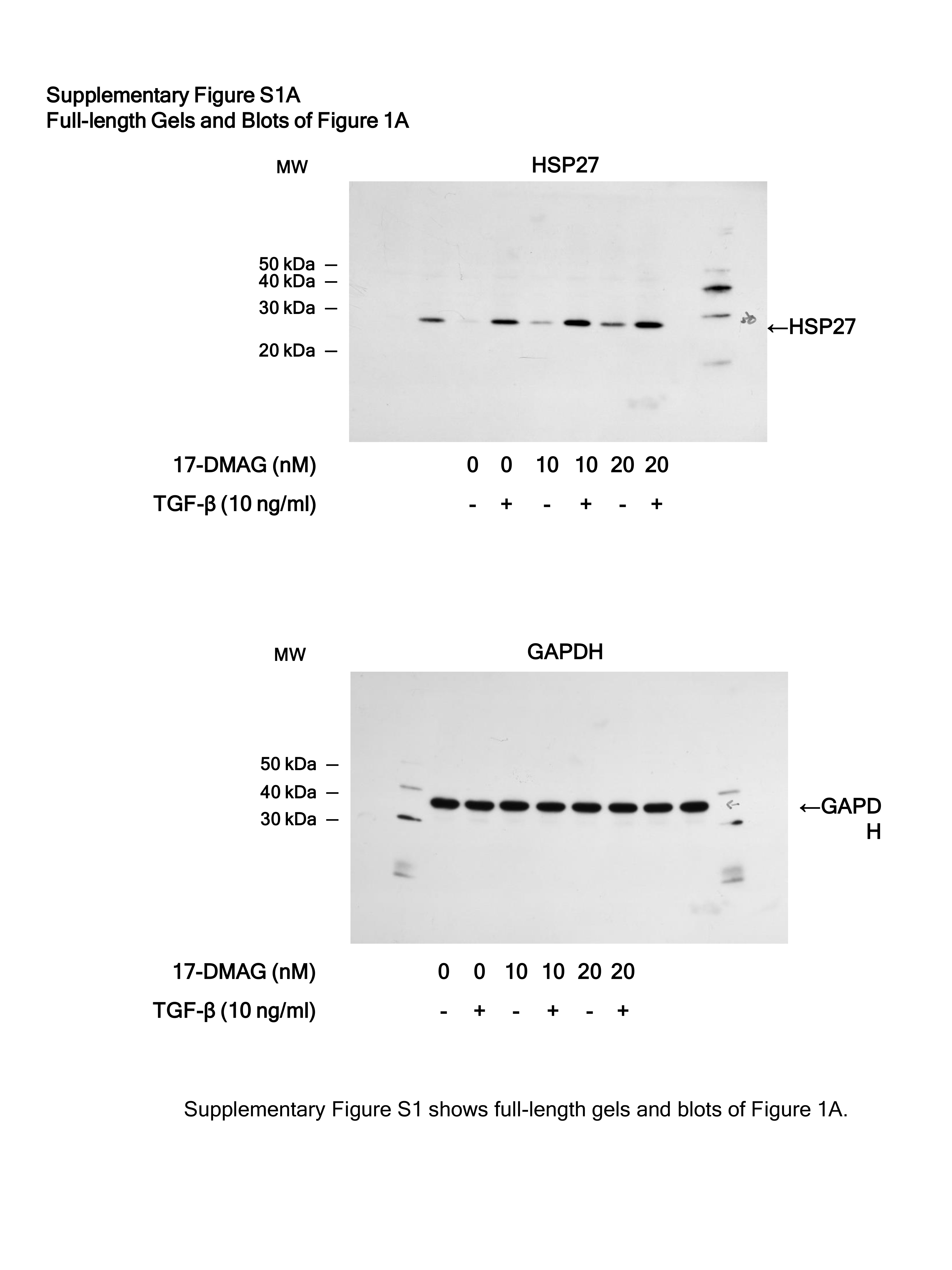

Supplement: Supplementary file 1 — Additional file 1 . Figure S1A, S1B, S2A, S2B, S3A, S3B, S3C, S4A, S4B, S5A, S5B, S6A and S6B show full-length gels and blots of Figure 1A, B, 2A 2B, 3A, 3B, 3C, 4A, 4B, 5A, 5B, 6A and 6B, respectively. [file 12891_2022_5419_MOESM1_ESM.zip › Supplementary Figure S1A - Full-length Gels and Blots of Figure 1A.tif]

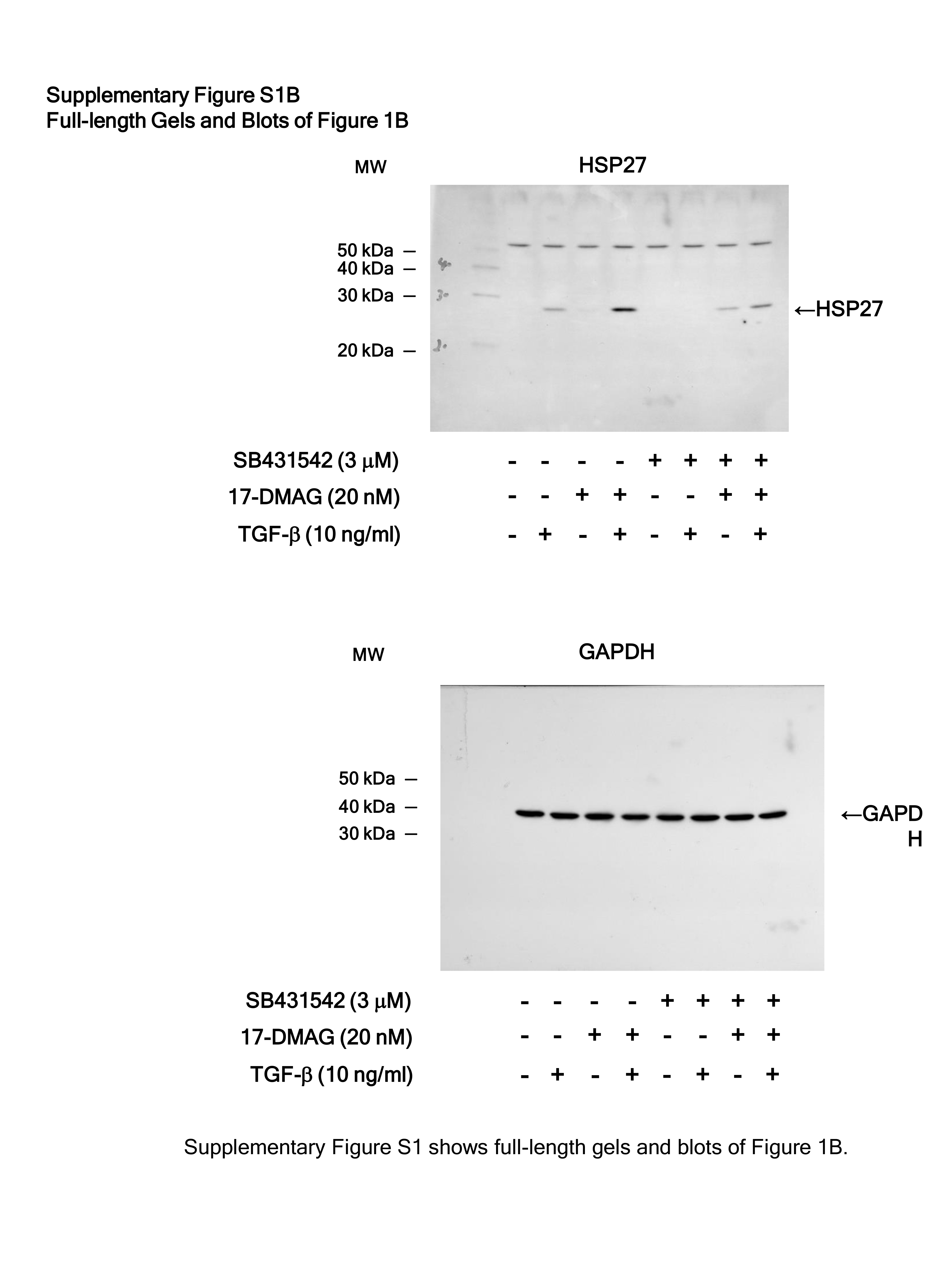

Supplement: Supplementary file 1 — Additional file 1 . Figure S1A, S1B, S2A, S2B, S3A, S3B, S3C, S4A, S4B, S5A, S5B, S6A and S6B show full-length gels and blots of Figure 1A, B, 2A 2B, 3A, 3B, 3C, 4A, 4B, 5A, 5B, 6A and 6B, respectively. [file 12891_2022_5419_MOESM1_ESM.zip › Supplementary Figure S1B - Full-length Gels and Blots of Figure 1B.tif]

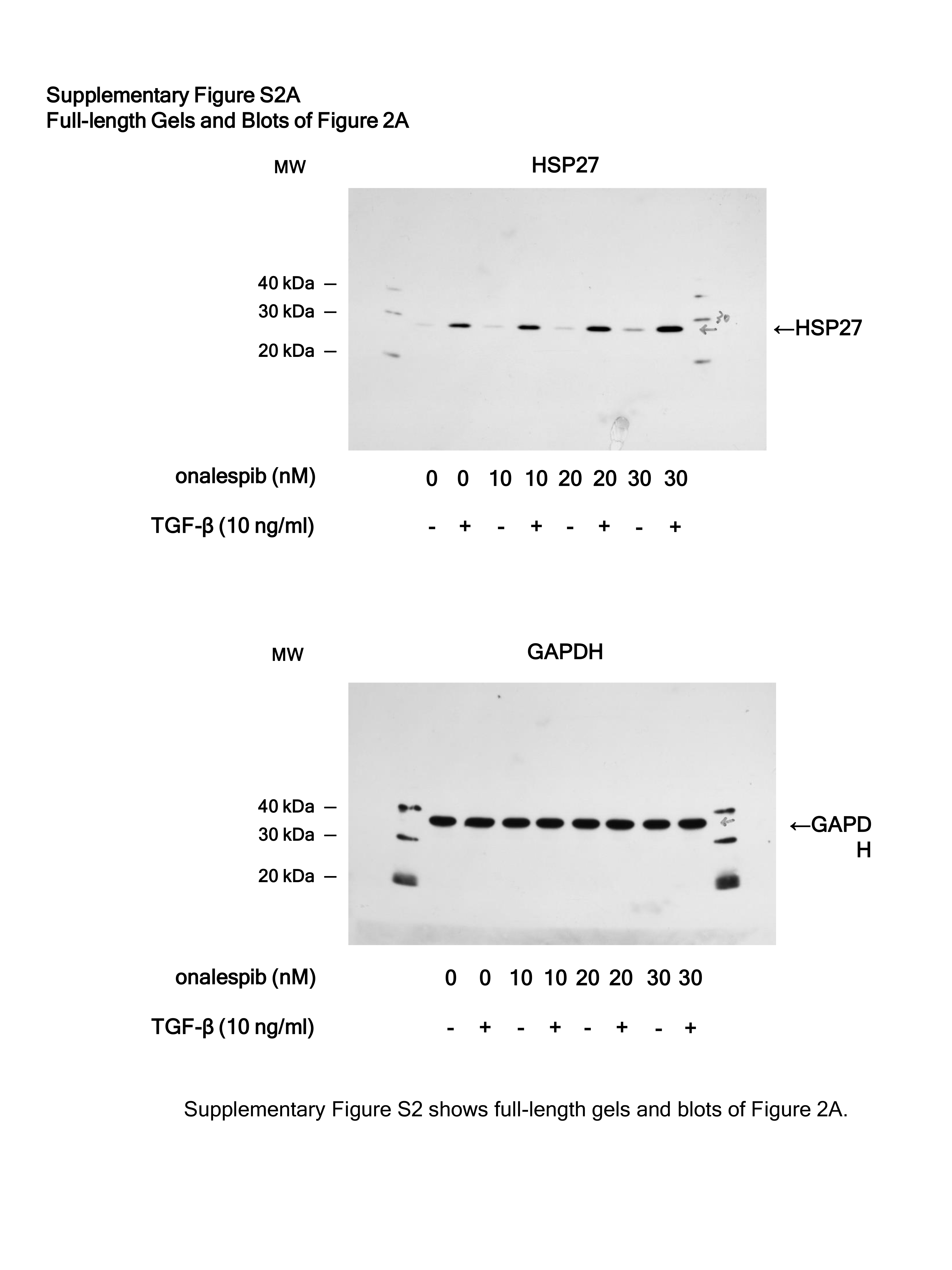

Supplement: Supplementary file 1 — Additional file 1 . Figure S1A, S1B, S2A, S2B, S3A, S3B, S3C, S4A, S4B, S5A, S5B, S6A and S6B show full-length gels and blots of Figure 1A, B, 2A 2B, 3A, 3B, 3C, 4A, 4B, 5A, 5B, 6A and 6B, respectively. [file 12891_2022_5419_MOESM1_ESM.zip › Supplementary Figure S2A - Full-length Gels and Blots of Figure 2A.tif]

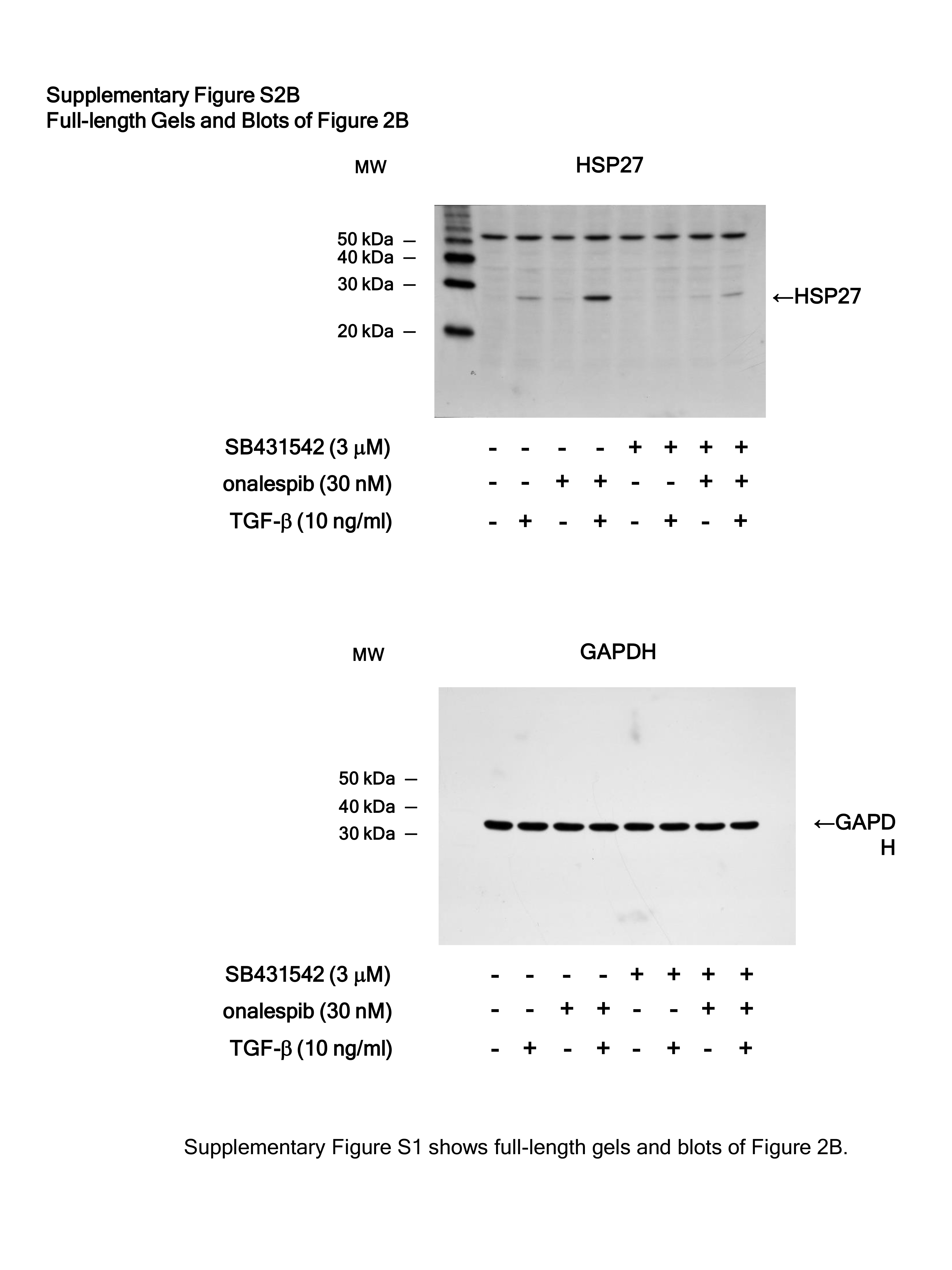

Supplement: Supplementary file 1 — Additional file 1 . Figure S1A, S1B, S2A, S2B, S3A, S3B, S3C, S4A, S4B, S5A, S5B, S6A and S6B show full-length gels and blots of Figure 1A, B, 2A 2B, 3A, 3B, 3C, 4A, 4B, 5A, 5B, 6A and 6B, respectively. [file 12891_2022_5419_MOESM1_ESM.zip › Supplementary Figure S2B - Full-length Gels and Blots of Figure 2B.tif]

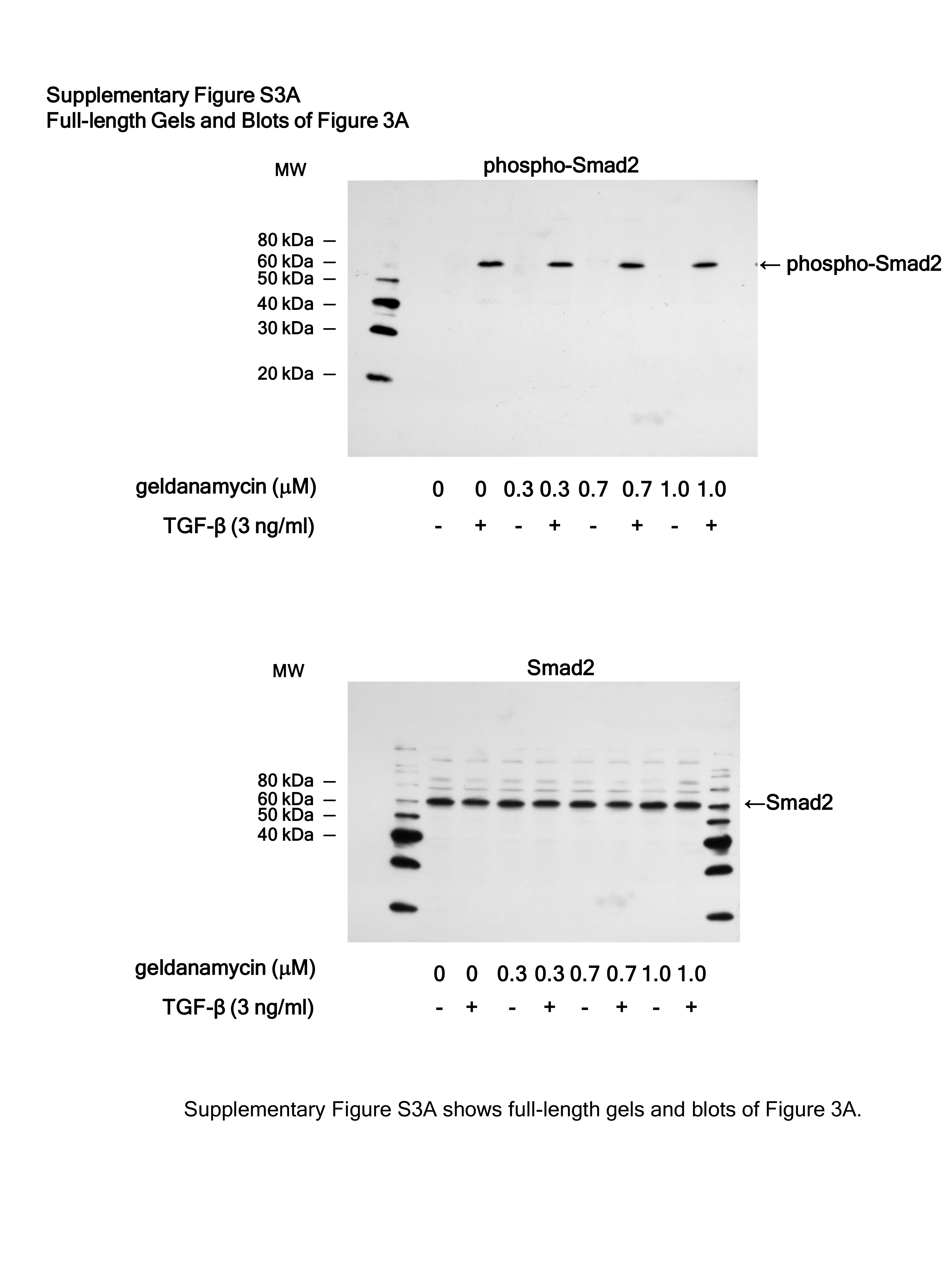

Supplement: Supplementary file 1 — Additional file 1 . Figure S1A, S1B, S2A, S2B, S3A, S3B, S3C, S4A, S4B, S5A, S5B, S6A and S6B show full-length gels and blots of Figure 1A, B, 2A 2B, 3A, 3B, 3C, 4A, 4B, 5A, 5B, 6A and 6B, respectively. [file 12891_2022_5419_MOESM1_ESM.zip › Supplementary Figure S3A - Full-length Gels and Blots of Figure 3A.tif]

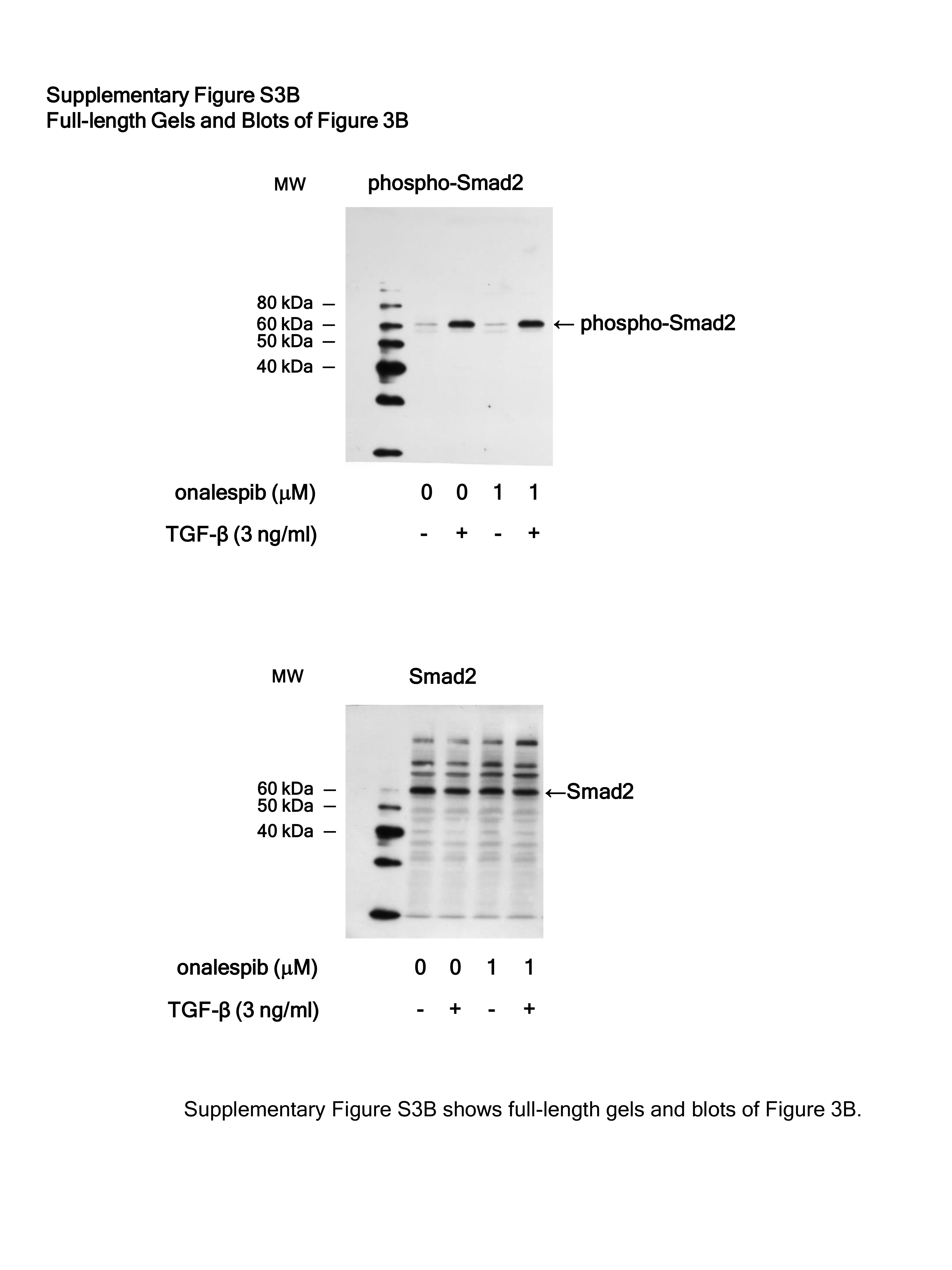

Supplement: Supplementary file 1 — Additional file 1 . Figure S1A, S1B, S2A, S2B, S3A, S3B, S3C, S4A, S4B, S5A, S5B, S6A and S6B show full-length gels and blots of Figure 1A, B, 2A 2B, 3A, 3B, 3C, 4A, 4B, 5A, 5B, 6A and 6B, respectively. [file 12891_2022_5419_MOESM1_ESM.zip › Supplementary Figure S3B - Full-length Gels and Blots of Figure 3B.tif]

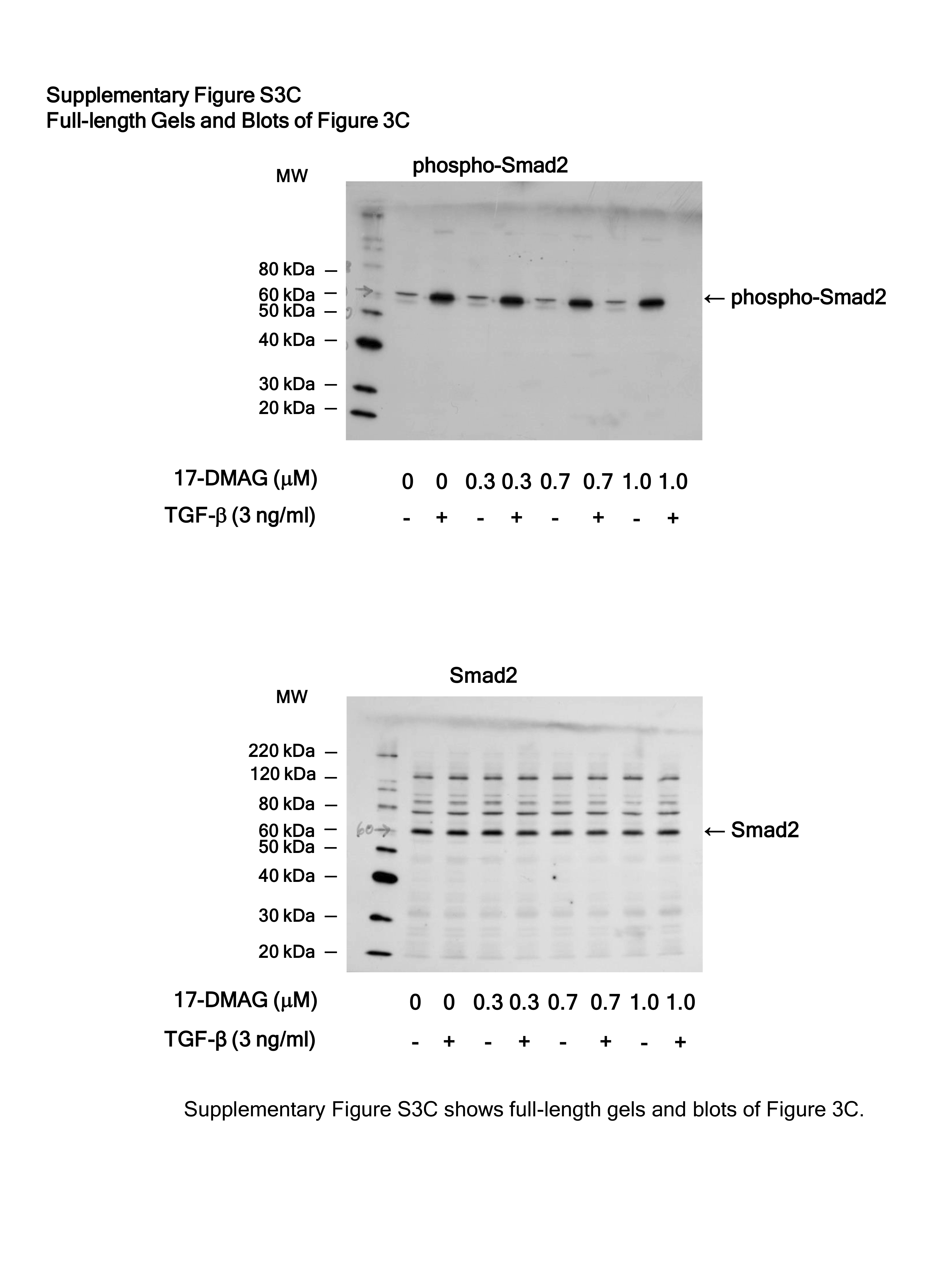

Supplement: Supplementary file 1 — Additional file 1 . Figure S1A, S1B, S2A, S2B, S3A, S3B, S3C, S4A, S4B, S5A, S5B, S6A and S6B show full-length gels and blots of Figure 1A, B, 2A 2B, 3A, 3B, 3C, 4A, 4B, 5A, 5B, 6A and 6B, respectively. [file 12891_2022_5419_MOESM1_ESM.zip › Supplementary Figure S3C - Full-length Gels and Blots of Figure 3C.tif]

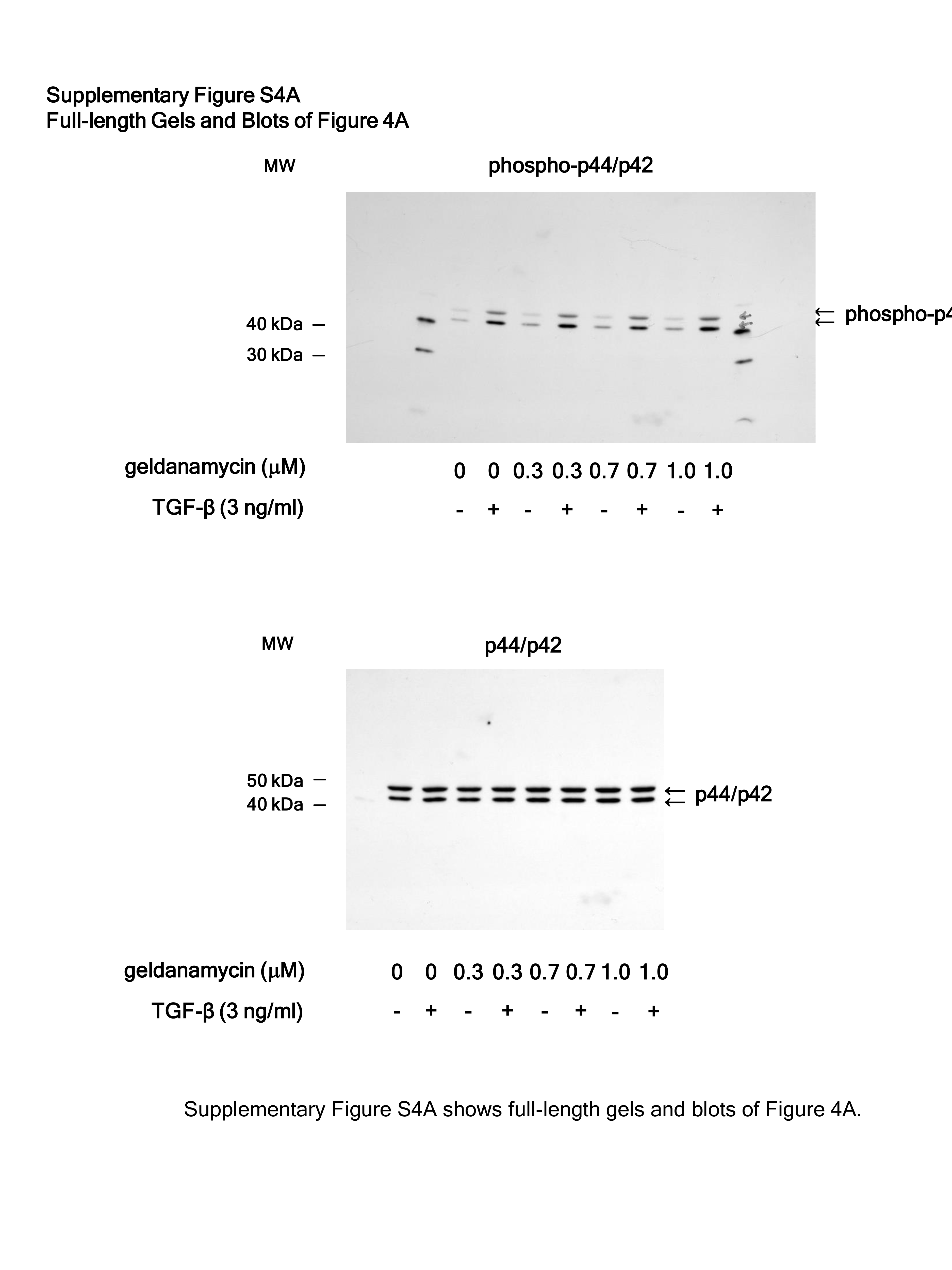

Supplement: Supplementary file 1 — Additional file 1 . Figure S1A, S1B, S2A, S2B, S3A, S3B, S3C, S4A, S4B, S5A, S5B, S6A and S6B show full-length gels and blots of Figure 1A, B, 2A 2B, 3A, 3B, 3C, 4A, 4B, 5A, 5B, 6A and 6B, respectively. [file 12891_2022_5419_MOESM1_ESM.zip › Supplementary Figure S4A - Full-length Gels and Blots of Figure 4A.tif]

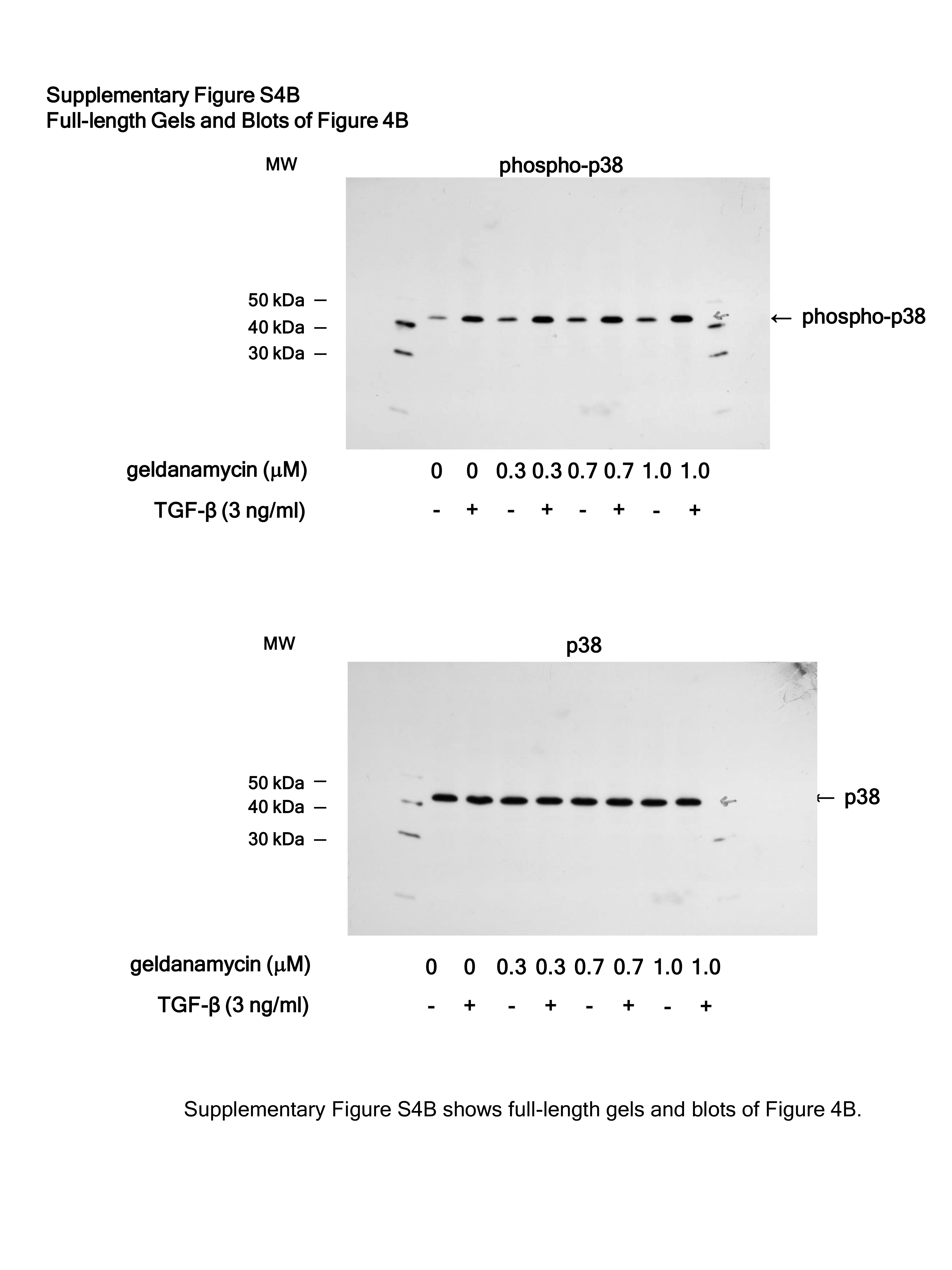

Supplement: Supplementary file 1 — Additional file 1 . Figure S1A, S1B, S2A, S2B, S3A, S3B, S3C, S4A, S4B, S5A, S5B, S6A and S6B show full-length gels and blots of Figure 1A, B, 2A 2B, 3A, 3B, 3C, 4A, 4B, 5A, 5B, 6A and 6B, respectively. [file 12891_2022_5419_MOESM1_ESM.zip › Supplementary Figure S4B - Full-length Gels and Blots of Figure 4B.tif]

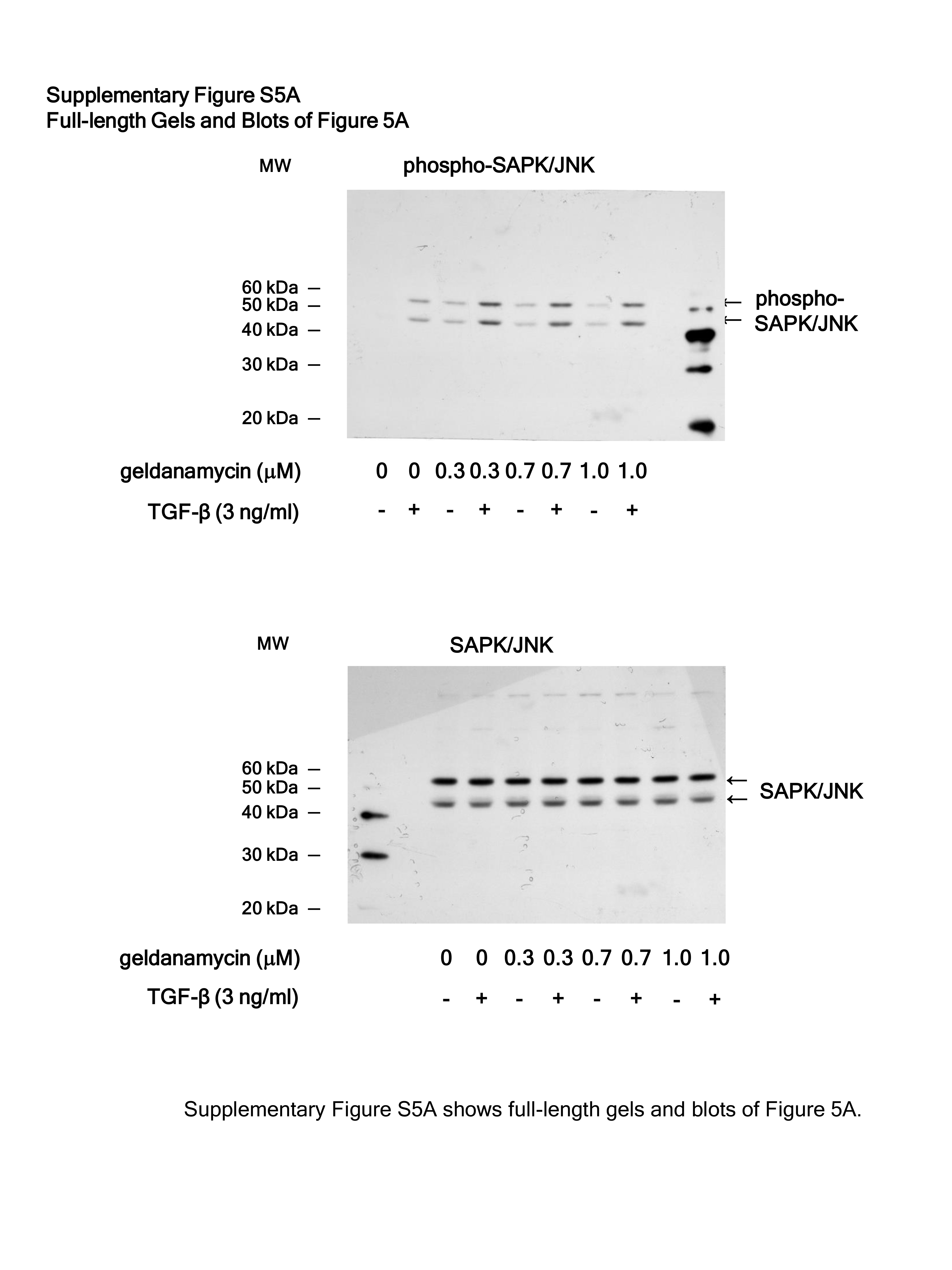

Supplement: Supplementary file 1 — Additional file 1 . Figure S1A, S1B, S2A, S2B, S3A, S3B, S3C, S4A, S4B, S5A, S5B, S6A and S6B show full-length gels and blots of Figure 1A, B, 2A 2B, 3A, 3B, 3C, 4A, 4B, 5A, 5B, 6A and 6B, respectively. [file 12891_2022_5419_MOESM1_ESM.zip › Supplementary Figure S5A - Full-length Gels and Blots of Figure 5A.tif]

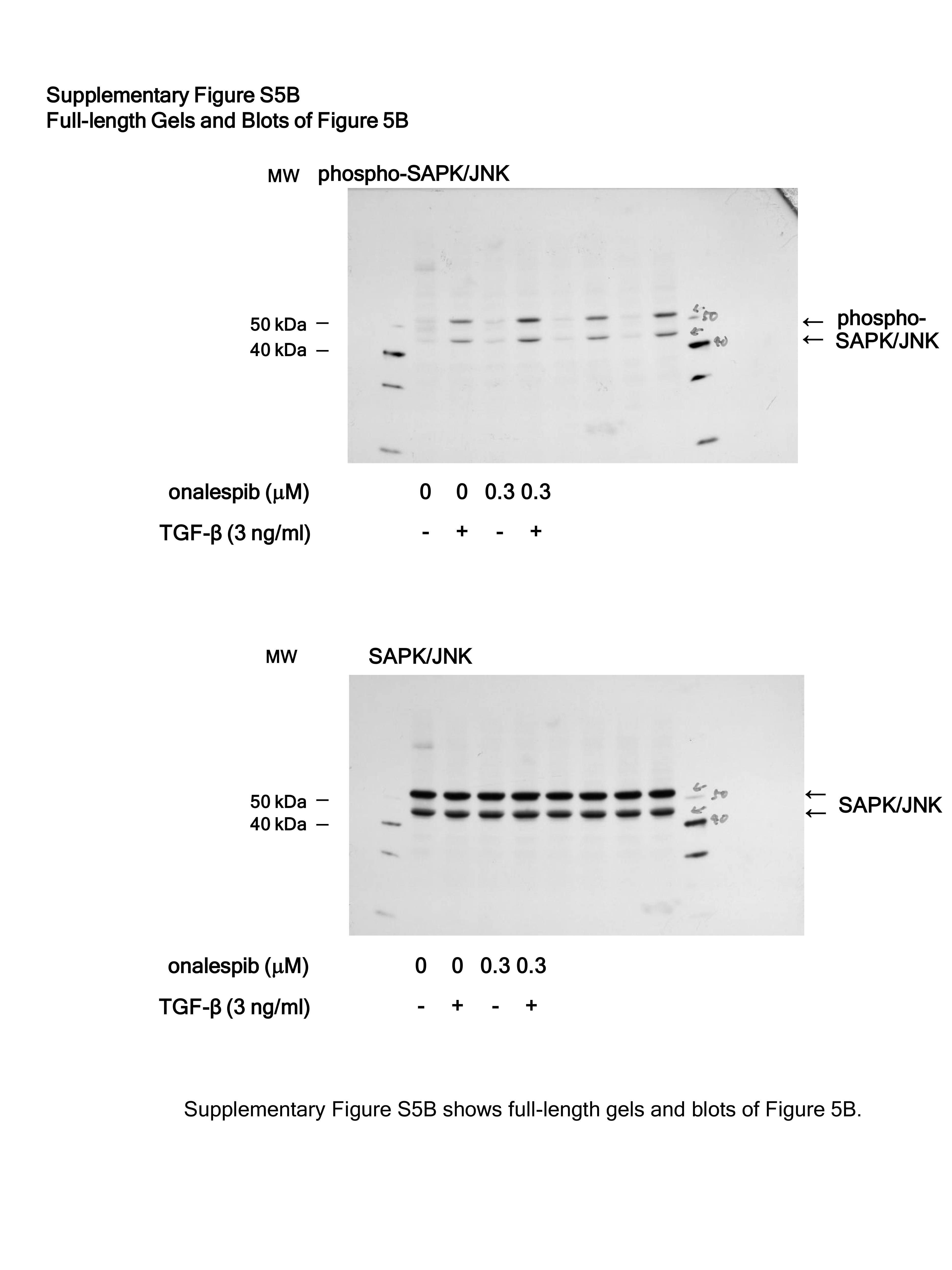

Supplement: Supplementary file 1 — Additional file 1 . Figure S1A, S1B, S2A, S2B, S3A, S3B, S3C, S4A, S4B, S5A, S5B, S6A and S6B show full-length gels and blots of Figure 1A, B, 2A 2B, 3A, 3B, 3C, 4A, 4B, 5A, 5B, 6A and 6B, respectively. [file 12891_2022_5419_MOESM1_ESM.zip › Supplementary Figure S5B - Full-length Gels and Blots of Figure 5B.tif]

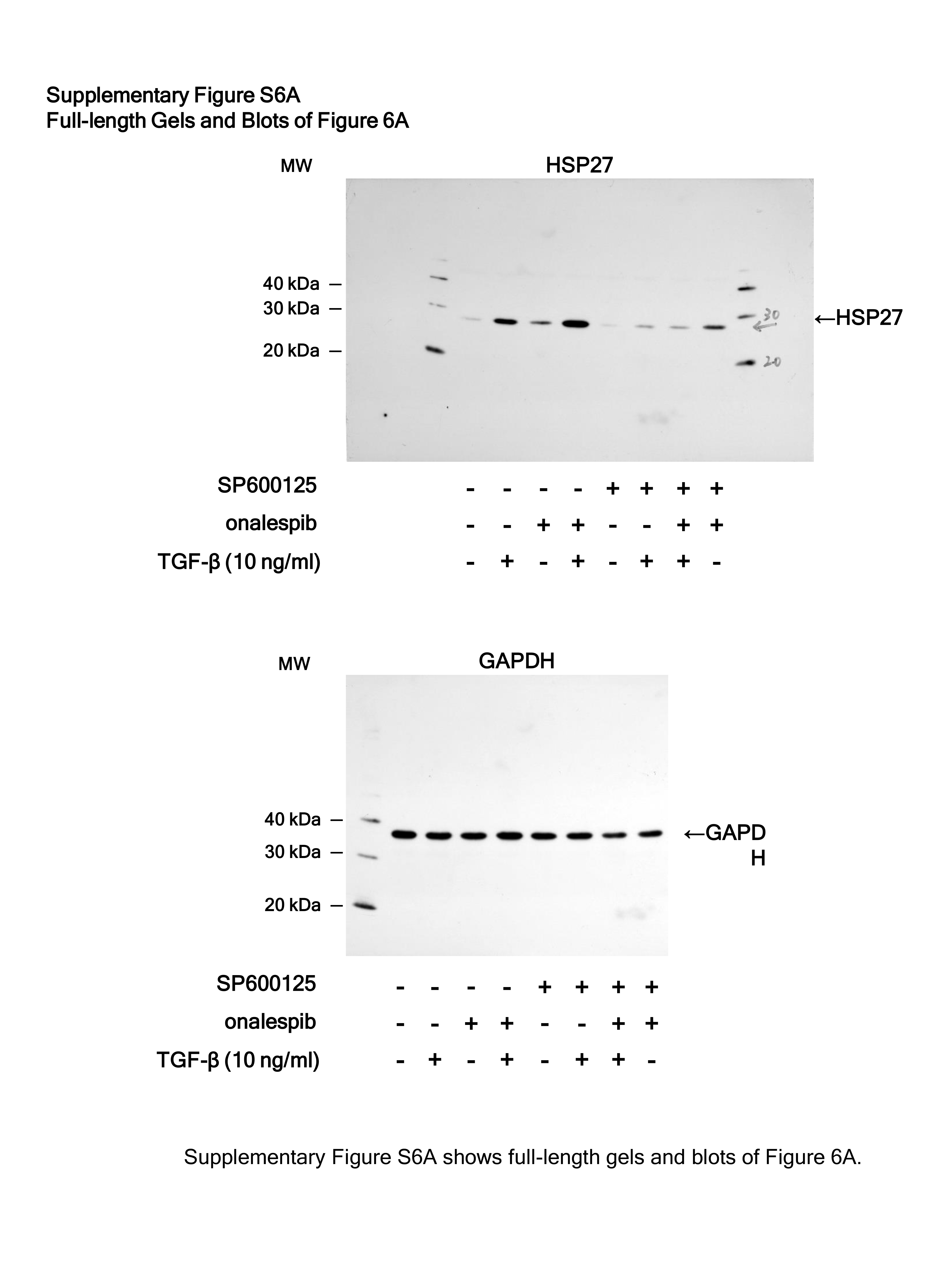

Supplement: Supplementary file 1 — Additional file 1 . Figure S1A, S1B, S2A, S2B, S3A, S3B, S3C, S4A, S4B, S5A, S5B, S6A and S6B show full-length gels and blots of Figure 1A, B, 2A 2B, 3A, 3B, 3C, 4A, 4B, 5A, 5B, 6A and 6B, respectively. [file 12891_2022_5419_MOESM1_ESM.zip › Supplementary Figure S6A - Full-length Gels and Blots of Figure 6A.tif]

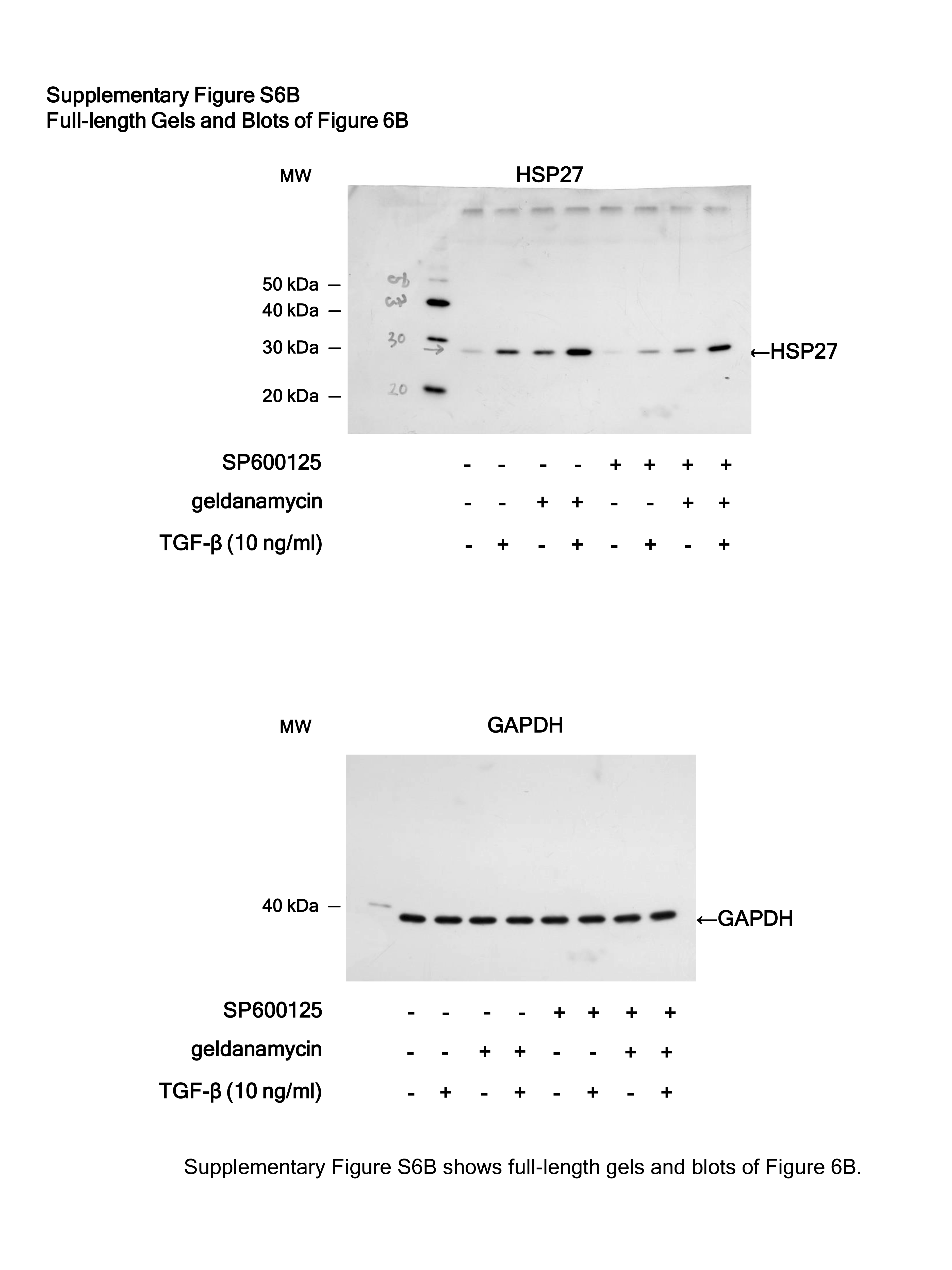

Supplement: Supplementary file 1 — Additional file 1 . Figure S1A, S1B, S2A, S2B, S3A, S3B, S3C, S4A, S4B, S5A, S5B, S6A and S6B show full-length gels and blots of Figure 1A, B, 2A 2B, 3A, 3B, 3C, 4A, 4B, 5A, 5B, 6A and 6B, respectively. [file 12891_2022_5419_MOESM1_ESM.zip › Supplementary Figure S6B - Full-length Gels and Blots of Figure 6B.tif]
